# Quantitative chemical proteomics reveals that phenethyl isothiocyanate covalently targets BID to promote apoptosis

**DOI:** 10.1038/s41420-024-02225-7

**Published:** 2024-10-29

**Authors:** Xiaoshu Dong, Xinqian Yu, Minghao Lu, Yaxin Xu, Liyan Zhou, Tao Peng

**Affiliations:** State Key Laboratory of Chemical Oncogenomics, School of Chemical Biology and Biotechnology, Peking University Shenzhen Graduate School, Shenzhen, 518055 China

**Keywords:** Target identification, Proteomics, Mechanism of action

## Abstract

Naturally occurring isothiocyanates (ITCs) found in cruciferous vegetables, such as benzyl isothiocyanate (BITC), phenethyl isothiocyanate (PEITC), and sulforaphane (SFN), have attracted significant research interest for their promising anti-cancer activity in vitro and in vivo. While the induction of apoptosis is recognized to play a key role in the anti-cancer effects of ITCs, the specific protein targets and associated upstream events underlying ITC-induced apoptosis remain unknown. In this study, we present a set of chemical probes that are derived from BITC, PEITC, and SFN and equipped with bioorthogonal alkynyl handles to systematically profile the target proteins of ITCs in live cancer cells. Using a competition-based quantitative chemical proteomics approach, we identify a range of candidate target proteins of ITCs enriched in biological processes such as apoptosis. We show that BID, an apoptosis regulator of the Bcl-2 family, is covalently modified by ITCs on its *N*-terminal cysteines. Functional characterization demonstrates that covalent binding to *N*-terminal cysteines of BID by PEITC results in conformational changes of the protein and disruption of the self-inhibitory interaction between *N*- and *C*-terminal regions of BID, thus unleashing the highly active *C*-terminal segment to exert downstream pro-apoptotic effects. Consistently, PEITC promotes the cleavage and mitochondrial translocation of BID, leading to a strong induction of apoptosis. We further show that mutation of *N*-terminal cysteines impairs the *N*- and *C*-terminal interaction of BID, relieving the self-inhibition and enhancing its apoptotic activity. Overall, our chemical proteomics profiling and functional studies not only reveal BID as the principal target of PEITC in mediating upstream events for the induction of apoptosis, but also uncover a novel molecular mechanism involving *N*-terminal cysteines within the first helix of BID in regulating its pro-apoptotic potential.

## Introduction

Isothiocyanates (ITCs) are a family of naturally occurring bioactive small molecules generated in cruciferous vegetables, including broccoli, cabbage, and cauliflower [1, 2]. Representative examples include benzyl isothiocyanate (BITC), phenethyl isothiocyanate (PEITC), and sulforaphane (SFN), all of which exhibit diverse biological activities [3] such as anti-cancer [4], anti-inflammation [5], and anti-microbial [6]. In particular, ITCs can potently inhibit the growth of many types of cancer in vitro and in vivo [4, 7]. ITCs and related formulations are being tested in clinical trials for cancer treatment [8, 9]. Studies have shown that apoptosis represents an important mechanism by which ITCs suppress the proliferation of cancer cells and inhibit tumorigenesis [4, 10, 11]. ITC-induced apoptosis may involve many pathways such as caspase activation [12], kinase signaling [13], mitochondrial dysfunction [14, 15], oxidative stress [16, 17], and glutathione metabolism [18, 19]. Nevertheless, the exact molecular basis of apoptosis induced by ITCs is still unclear.

Owing to the unique electrophilicity and chemical reactivity, ITCs can covalently modify protein targets to exert their biological effects [20, 21]. For example, ITCs directly modify the *N*-terminal proline of macrophage migration inhibitory factor (MIF) to abolish its tautomerase activity [22, 23]. Understanding the protein target profiles of ITCs could thus provide invaluable insights into their mechanisms of action for inducing apoptosis [24]. Over the years, a handful of studies have explored the efforts to identify binding targets of ITCs using ITC-derived probes and proteomic analysis [25]. Early studies developed radioisotope-labeled ITCs [26, 27], biotin-containing ITC probes [22, 28], and ITC-functionalized agarose resins [23], which are however limited in radioactive detection or cell lysate applications. The advances of bioorthogonal chemistry [29] have facilitated the development of PEITC-derived and SFN-mimic bioorthogonal chemical probes for readily profiling their target proteins in live cells [30–33]. However, these studies either only performed the proteomic analyses in noncancerous cells [30], or only identified potential targets without further functional characterization [32, 33]. In addition, a comprehensive comparison of target profiles of different ITCs has not been reported. More importantly, the direct target proteins and associated biochemical events underlying ITC-induced apoptosis remain to be elucidated.

Apoptosis is primarily regulated by the Bcl-2 protein family, which consists of anti-apoptotic members (e.g., Bcl-2 and Bcl-xL) and pro-apoptotic members including proteins with multiple Bcl-2 homology (BH) domains (e.g., BAX and BAK) and BH3-only proteins, such as BID and BAD [34]. As an abundant pro-apoptotic protein, BID is only weakly active but can be cleaved by caspase-8 to generate the strongly pro-apoptotic *C*-terminal fragment in response to apoptotic signaling [35]. This *C*-terminal fragment uses its BH3 domain not only to activate the downstream effectors to induce BAX/BAK-dependent mitochondrial membrane permeabilization, but also to neutralize the effects of anti-apoptotic proteins such as Bcl-xL [36, 37]. Although BH3 mimetics have been developed as potential apoptosis-inducing and cancer therapeutic agents [38], small molecules directly targeting BID have not been reported yet.

Herein, we report alkynyl-functionalized ITC-derived probes and quantitative chemical proteomics for comprehensively profiling the target proteins of BITC, PEITC, and SFN in cancer cells. We identified a series of candidate targets of ITCs involved in biological processes such as apoptosis and chose one of them, i.e., BID, for functional characterization. The results show that PEITC covalently targets the *N*-terminal cysteines of BID, which disrupts the self-inhibitory interaction between *N*- and *C*-terminal regions of BID to unleash its pro-apoptotic activity and promote apoptosis. We further demonstrate that *N*-terminal cysteines of BID are essential for maintaining the self-inhibitory *N*- and *C*-terminal interaction of BID. Our studies thus directly link PEITC-mediated covalent modifications of BID to PEITC-induced apoptosis and reveal a novel autoinhibitory mechanism for regulating the pro-apoptotic potential of BID.

## Results

### Alkynyl-functionalized ITC probes label the target proteins of ITCs in live cells

**Fig. 1:**
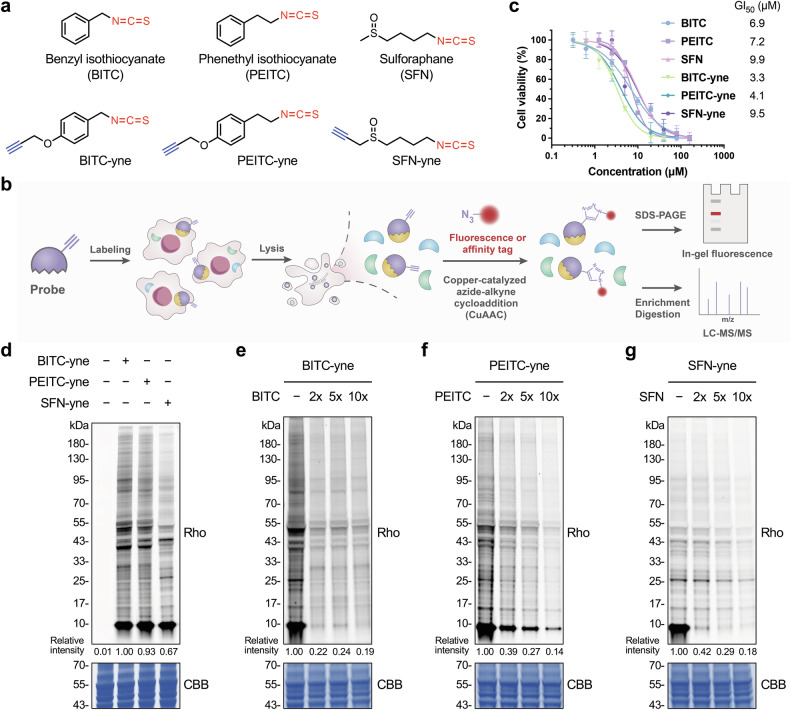
Development of chemical probes for labeling target proteins of ITCs in live cells. **a** Structures of representative natural ITCs (i.e., BITC, PEITC, and SFN) and ITC-derived chemical probes (i.e., BITC-yne, PEITC-yne, and SFN-yne). **b** Schematic for profiling the target proteins of ITCs with ITC-derived probes. Cells are labeled with ITC-derived probes, lysed, and subjected to CuAAC reactions with fluorescence and affinity tags for in-gel fluorescence and LC-MS/MS analyses, respectively. **c** Dose-dependent inhibition of cell growth by natural ITCs and ITC-derived probes. MCF-7 cells were incubated with natural ITCs or ITC probes for 72 h and measured for viability. Data are shown as mean ± sd (*n* = 6). GI_50_ values were calculated as the concentrations for half of maximal inhibition of cell growth. **d** In-gel fluorescence analysis of proteins labeled by ITC probes. **e** In-gel fluorescence analysis of proteins labeled by BITC-yne with dose-dependent BITC competition. **f** In-gel fluorescence analysis of proteins labeled by PEITC-yne with dose-dependent PEITC competition. **g** In-gel fluorescence analysis of proteins labeled by SFN-yne with dose-dependent SFN competition. MCF-7 cells were labeled with the probe (20 µM) for 0.5 h and lysed for conjugation with azido-rhodamine and in-gel fluorescence analysis. For competition assays in (**e**–**g**), MCF-7 cells were pre-treated with the natural ITC at indicated doses (shown as fold of the probe concentration) for 0.5 h and labeled with the probe (5 µM) for 0.5 h. Relative rhodamine intensities were quantified and shown. Rho: rhodamine; CBB: Coomassie Brilliant Blue staining.

To profile the target proteins of ITCs, we focused on three representative natural ITCs, including BITC, PEITC, and SFN, and designed respective alkynyl-functionalized ITC probes, i.e., BITC-yne, PEITC-yne, and SFN-yne (Fig. 1a). These probes feature minimal structural perturbations, retain the intact ITC functionality for covalently labeling the target proteins in live cells, and contain bioorthogonal alkynyl handles for conjugating with fluorescence and affinity tags through Cu(I)-catalyzed azide-alkyne cycloaddition (CuAAC) [39] reactions for in-gel fluorescence and proteomics analyses, respectively (Fig. 1b). These ITC probes were synthesized *via* routine operations of organic chemistry, as detailed in Supplementary Information.

Before utilizing these probes for protein labeling, we sought to validate that they retain the biological activities of natural ITCs. We first examined their anti-proliferation activities in MCF-7 human breast cancer cells, as current clinical trials on ITCs are mostly focused on breast cancer [9]. BITC-yne, PEITC-yne, and SFN-yne exhibited marked cell growth inhibitory activities (Fig. 1c), though with some variation in potency compared to their natural counterparts. A similar difference, i.e., the stronger cytotoxicity of the alkynyl derivative of PEITC compared to PEITC, has been observed in previous studies [32], although the underlying mechanisms remain unclear. Further cell cycle analyses demonstrated that BITC-yne and PEITC-yne, like their natural counterparts [26, 40, 41], induced accumulation of G2/M phase cells (Supplementary Fig. S1), a well-documented effect of ITCs leading to cell cycle arrest. SFN and SFN-yne were less potent in inducing G2/M accumulation, consistent with previous studies [26]. Therefore, these results confirm that the ITC probes largely preserve the key biological activities of their natural counterparts and can potentially serve as functional probes for profiling the target proteins of ITCs.

We then investigated the ability of ITC probes to label proteins in live cells. MCF-7 cells were incubated with individual probes and lysed for CuAAC reactions with azido-rhodamine and in-gel fluorescence analysis. The results showed that a range of proteins were labeled by BITC-yne, PEITC-yne, and SFN-yne and that the labeling efficiency of BITC-yne and PEITC-yne was higher than that of SFN-yne (Fig. 1d). In-gel fluorescence signals of ITC probes were dependent on the probe concentration and labeling period, almost reaching saturation within half an hour (Supplementary Fig. S2). To determine the labeling specificity of ITC probes, we performed competition experiments in which cells were pre-treated with the natural ITC before probe incubation. The results demonstrated that BITC, PEITC, and SFN significantly diminished the labeling of BITC-yne, PEITC-yne, and SFN-yne, respectively, in a dose-dependent manner (Fig. 1e–g), suggesting that ITC probes share the same target profiles as natural ITCs. Cysteine alkylation agents, such as iodoacetamide (IAA) and *N*-ethylmaleimide (NEM), were much less efficient than natural ITCs in competing with ITC probes (Supplementary Fig. S3), indicating that ITC probes may target a distinct set of proteins from IAA and NEM. Collectively, these results demonstrate that alkynyl-functionalized ITC probes covalently label the target proteins of ITCs in live cells.

### Quantitative chemical proteomics profiles the target proteins of ITCs

**Fig. 2:**
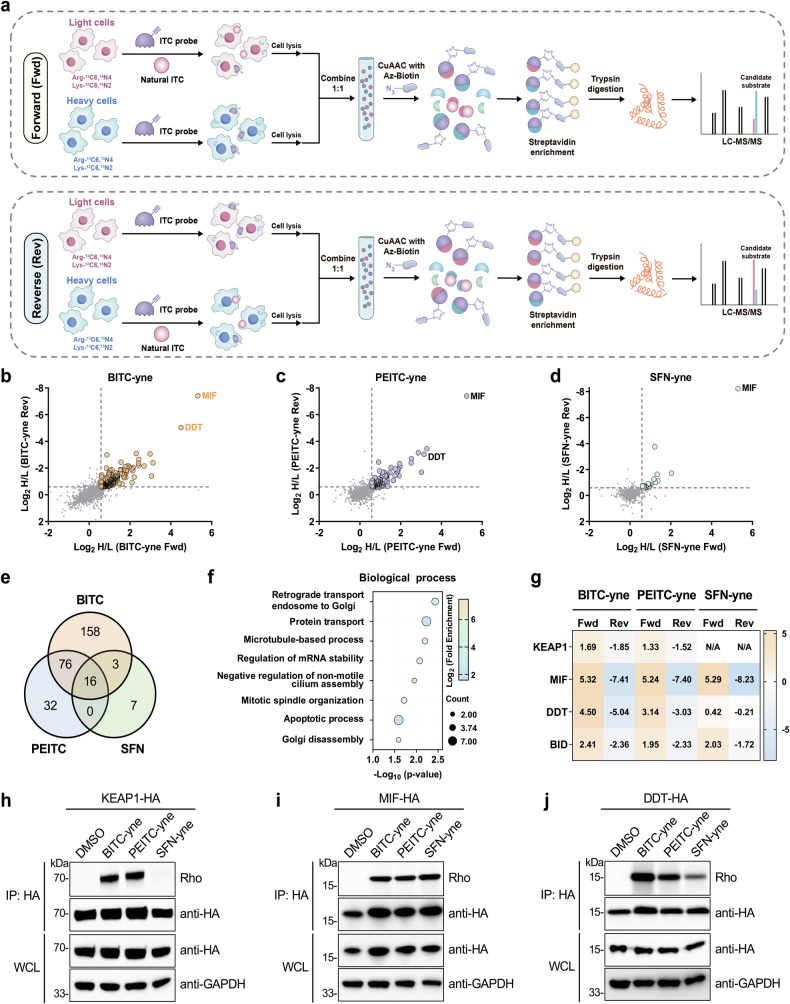
Quantitative chemical proteomics profiling of the target proteins of ITCs. **a** Workflow of dual SILAC experiments for profiling the target proteins of ITCs. In the Forward (Fwd) and Reverse (Rev) SILAC experiments, MCF-7 cells grown in heavy and light media were switched for competition with the natural ITC before probe labeling. Heavy and light cell lysates were combined, conjugated with azido-biotin, and subjected to enrichment and digestion for LC-MS/MS analysis. **b** Scatter plot of the SILAC quantitative chemical proteomics data obtained with BITC-yne. **c** Scatter plot of the SILAC quantitative chemical proteomics data obtained with PEITC-yne. **d** Scatter plot of the SILAC quantitative chemical proteomics data obtained with SFN-yne. H/L represents the heavy-to-light SILAC ratio between heavy and light isotope labels. **e** Venn diagram showing the numbers of candidate target proteins of BITC, PEITC, and SFN. **f** GO analysis of the candidate target proteins of any two ITCs in terms of biological processes. **g** SILAC ratios (with log_2_ transformation) of selected candidate target proteins (e.g., KEAP1, MIF, DDT, and BID) quantified in the quantitative chemical proteomics profiling experiments. **h** In-gel fluorescence validation of KEAP1 labeling by ITC probes. **i** In-gel fluorescence validation of MIF labeling by ITC probes. **j** In-gel fluorescence validation of DDT labeling by ITC probes. For (**h**–**j**), HEK293T cells were transfected to express the candidate target protein, labeled with the probe (20 µM) for 0.5 h, and subjected to immunoprecipitation (IP), followed by conjugation with azido-rhodamine and in-gel fluorescence analysis. Rho: rhodamine.

We then sought to identify the target proteins of ITCs in cancer cells using a competition-based quantitative chemical proteomics approach that integrates ITC probes and chemical proteomics [42] with stable-isotope labeling with amino acids in cell culture (SILAC) [43]. To enhance identification reliability, dual SILAC experiments [44] were performed (Fig. 2a). In the “Forward” analysis, MCF-7 cells grown in heavy media were labeled with the probe, whereas cells grown in light media were subjected to competition with the corresponding natural ITC. In the “Reverse” analysis, cells grown in light media were labeled with the probe, whereas cells grown in heavy media were subjected to competition with the corresponding natural ITC. Heavy and light cell lysates were combined and reacted with azido-biotin through CuAAC. After streptavidin enrichment and trypsin digestion, the resulting peptides were analyzed by liquid chromatography-tandem mass spectrometry (LC-MS/MS) for protein identification and quantification (Fig. 2a). Candidate target proteins of ITCs were expected to be quantified with high and low heavy-to-light (H/L) SILAC ratios in Forward and Reverse analyses, respectively. We carried out three sets of dual SILAC experiments using BITC-yne, PEITC-yne, and SFN-yne, and in total identified 253 candidate BITC targets, 124 candidate PEITC targets, and 26 candidate SFN targets (Supplementary Table S1), all of which met the cutoffs of log_2_H/L > 0.59 and log_2_H/L < −0.59 in Forward and Reverse analyses, respectively, in the corresponding dual SILAC experiment (Fig. 2b–d). A comparison of these candidate target proteins showed that BITC, PEITC, and SFN shared a subset of common target proteins with their unique targets (Fig. 2e). Gene ontology (GO) analysis on the target proteins of any two ITCs suggested that they were significantly enriched in diverse biological processes including apoptosis (Fig. 2f).

We selected several candidate target proteins for biochemical validation. In this regard, cells were transfected to express HA-tagged candidate proteins and labeled with ITC probes. The candidate proteins were immunoprecipitated, reacted with azido-rhodamine, and analyzed by in-gel fluorescence. Known targets of ITCs, such as KEAP1 [45, 46] and MIF [22, 23], were identified in the proteomic analyses and validated to be modified by ITC probes (Fig. 2g–i). Interestingly, we found that *D*-dopachrome tautomerase (DDT), a second member of the MIF family sharing 34% sequence identity and an overlapping functional spectrum with MIF [47], was also modified by ITC probes (Fig. 2g and j), suggesting that DDT should be considered for understanding the anti-inflammatory activity of ITCs. Moreover, three newly identified candidate target proteins, i.e., MAP4, TXN, and LSM14A, were all validated to be labeled by ITC probes (Supplementary Fig. S4). Together, these results confirm the reliability of our competition-based quantitative chemical proteomics for profiling the target proteins of ITCs.

### ITCs covalently modify *N*-terminal cysteines of BID

**Fig. 3:**
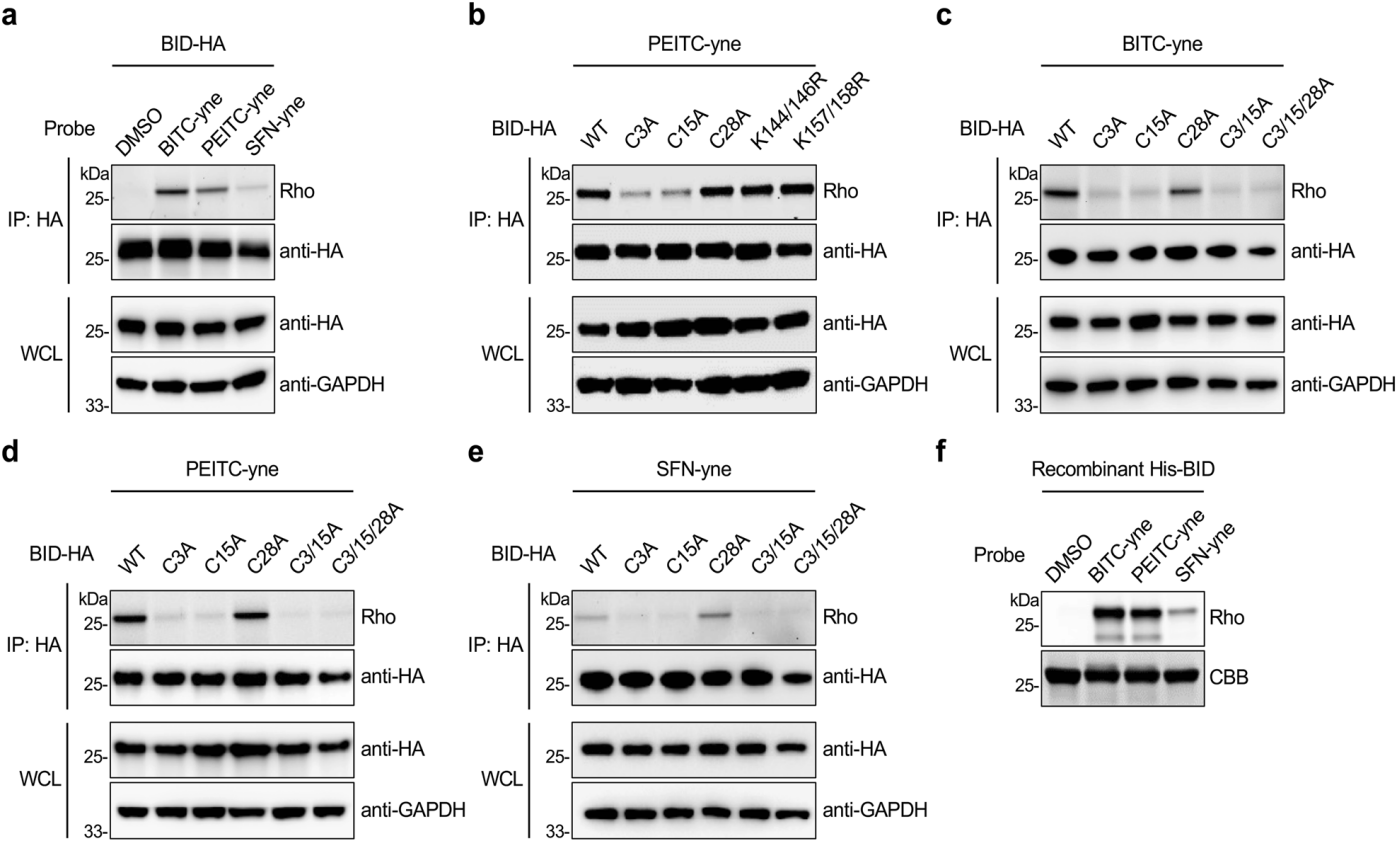
BID is covalently modified by ITC probes on *N*-terminal cysteines. **a** In-gel fluorescence validation of BID labeling by ITC probes. **b** Investigation of the modification sites in BID labeled by PEITC-yne. **c** Characterization of cysteine residues in BID that are modified by BITC-yne. **d** Characterization of cysteine residues in BID that are modified by PEITC-yne. **e** Characterization of cysteine residues in BID that are modified by SFN-yne. For (**a**–**e**), HEK293T cells were transfected to express BID or its mutants, labeled with the probe (20 µM) for 0.5 h, and subjected to immunoprecipitation (IP), followed by conjugation with azido-rhodamine and in-gel fluorescence analysis. **f** Labeling of recombinant BID by ITC probes in vitro. Purified BID protein was incubated with ITC probes (20 µM) for 2 h, conjugated with azido-rhodamine, and analyzed by in-gel fluorescence. Rho: rhodamine; CBB: Coomassie Brilliant Blue staining.

Notably, BID was identified as a candidate target of BITC, PEITC, and SFN in our proteomics analyses (Fig. 2g). In-gel fluorescence analyses confirmed that BID was strongly labeled by BITC-yne and PEITC-yne, but less modified by SFN-yne, in live cells (Fig. 3a). To map the modification sites, we examined nucleophilic cysteine and lysine residues of BID. There are three cysteines (i.e., C3, C15, and C28) in the *N*-terminus of BID and four lysines (i.e., K144, K146, K157, and K158) near the *C*-terminus. While mutation of lysines hardly affected BID labeling by PEITC-yne, the cysteine-to-alanine mutants showed decreased in-gel fluorescence signals (Fig. 3b). Particularly, mutation of C3 or C15 substantially reduced the labeling of BID by ITC probes and mutation of C28 slightly decreased the labeling of BITC-yne and PEITC-yne (Fig. 3c–e). The double mutant (i.e., C3/15 A) and triple mutant (i.e., C3/15/28 A; 3CA) showed almost completely diminished labeling (Fig. 3c–e), indicating that *N*-terminal cysteines of BID are the potential modification sites of ITCs. The cysteines of human BID are not conserved in mouse Bid that contains two cysteines, i.e., C30 at the *N*-terminus and C126 near the *C*-terminus. In line with this, mouse Bid was much less labeled by ITC probes than human BID (Supplementary Fig. S5).

Consistent with the cellular data, recombinant human BID prepared from *E. coli* was strongly labeled by BITC-yne and PEITC-yne in vitro, as well as by SFN-yne at lower efficiency (Fig. 3f). While the C3A, C15A, and C28A mutants were still labeled by ITC probes in vitro, the C3/15 A mutant was significantly less modified and the C3/15/28 A mutant was unmodified by ITC probes (Supplementary Fig. S6). To further verify that ITCs directly modify cysteines, PEITC-treated recombinant BID was digested with trypsin and analyzed by LC-MS/MS. The MS/MS spectra demonstrated that three cysteines of BID were indeed covalently modified by PEITC to form dithiocarbamate adducts (Supplementary Fig. S7). Collectively, these data suggest that ITCs promiscuously and covalently target *N*-terminal cysteines of BID.

### ITCs induce the cleavage and mitochondrial localization of BID and promote apoptosis

**Fig. 4:**
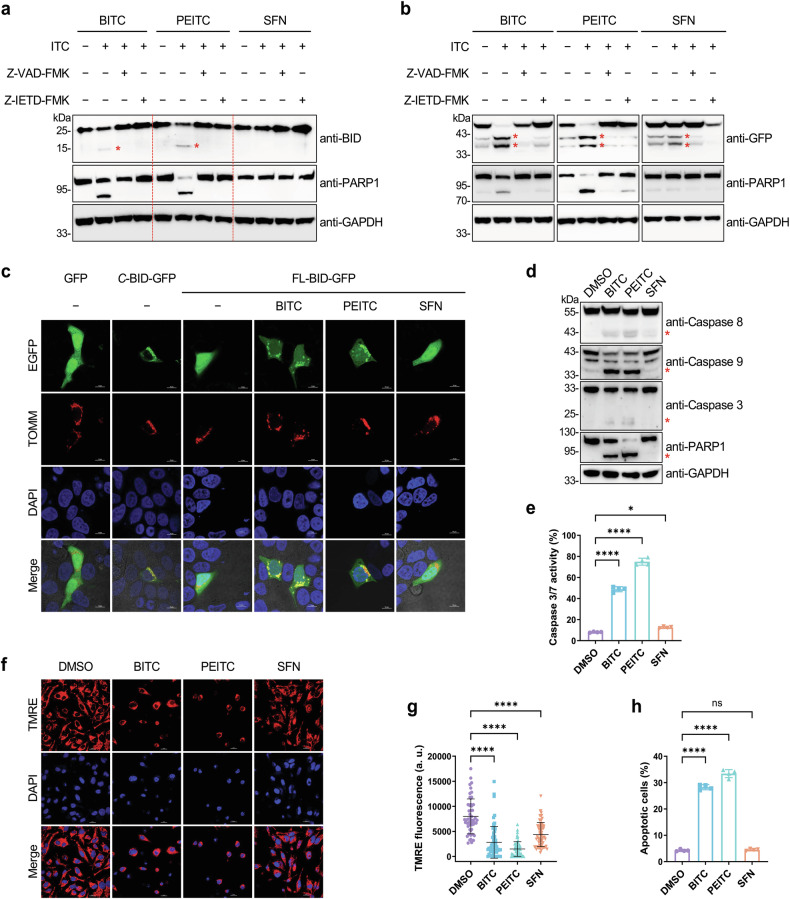
ITCs induce the cleavage of BID and promote apoptosis. **a** Western blotting analysis of endogenous BID cleavage induced by ITCs. HeLa cells were incubated with ITCs (20 µM) for 4 h in the absence or presence of Z-VAD-FMK (20 µM) or Z-IETD-FMK (20 µM). **b** Western blotting analysis of exogenous BID cleavage induced by ITCs. HeLa cells expressing GFP-tagged BID were incubated with ITCs (20 µM) for 4 h in the absence or presence of Z-VAD-FMK (20 µM) or Z-IETD-FMK (20 µM). Red asterisks indicate the cleaved proteins. **c** Confocal fluorescence imaging of BID. HeLa cells expressing GFP-tagged *C*-BID or full-length BID (FL-BID) and TOMM-mCherry were treated with ITCs (20 µM) as indicated for 4 h and imaged by confocal fluorescence microscopy. Scale bars = 10 µm. **d** Western blotting analysis of caspases after treatment with ITCs. HeLa cells were treated with ITCs (20 µM) as indicated for 4 h. Red asterisks indicate the cleaved proteins. **e** Caspase-3/7 activity measured by a caspase-3 substrate and flow cytometry. HeLa cells were treated with ITCs (20 µM) for 4 h. Data are shown as mean ± sd (*n* = 4). **f** Fluorescence imaging of mitochondrial membrane potential using TMRE in HeLa cells treated with ITCs (20 µM) for 4 h. Scale bars = 20 µm. **g** Quantification of fluorescence imaging data shown in (**f**). Data are shown as mean ± sd (*n* = 50 cells per condition). **h** Percentages of apoptotic cells measured by Annexin V staining and flow cytometry. HeLa cells were treated with ITCs (20 µM) for 4 h. Data are shown as mean ± sd (*n* = 4). Statistical analyses in (**e**, **g**, **h**) were performed with one-way ANOVA test (*p < 0.05, ****p < 0.0001, and ns *p* > 0.05).

Considering the well-known role of BID in apoptosis, we next explored the effects of ITCs on triggering BID cleavage and inducing apoptosis. Our initial examination indicated that BITC and PEITC, and to a lesser extent SFN, induced BID cleavage and apoptosis in MCF-7 cells (Supplementary Fig. S8). To validate these effects in a different cellular context, we conducted a detailed investigation using HeLa cells since ITC-induced apoptosis was first demonstrated in these cells [12]. Given our interest in understanding the upstream events leading to apoptosis, we focused on early time points following ITC treatment (i.e., 4 h). Western blotting analyses showed that both BITC and PEITC, but not SFN, induced the cleavage of endogenous BID, which could be suppressed by pan-caspase inhibitor Z-VAD-FMK or selective caspase-8 inhibitor Z-IETD-FMK (Fig. 4a). Similarly, BITC and PEITC induced the cleavage of overexpressed BID in a caspase-8-dependent manner more efficiently than SFN (Fig. 4b and Supplementary Fig. S9). Fluorescence imaging experiments demonstrated that treatment of cells with BITC or PEITC, but not SFN, led to the translocation of BID to mitochondria, where the *C*-terminal fragment (*C*-BID) is localized (Fig. 4c). Inhibition of caspase activities blocked PEITC-induced BID translocation to mitochondria (Supplementary Fig. S10), indicating that BID cleavage by caspases is required for its mitochondrial localization. In line with these results, the cleaved pro-caspases (e.g., caspase-8, caspase-9, and caspase-3) and PARP1, hallmarks of apoptosis [48], were increased after treatment of cells with BITC or PEITC (Fig. 4d). Likewise, the activity of caspase-3/7 was substantially elevated in BITC- or PEITC-treated cells (Fig. 4e). In addition, the mitochondrial membrane potential measured by tetramethylrhodamine ethyl ester (TMRE) fluorescence was greatly reduced in cells treated with BITC or PEITC, but less decreased in SFN-treated cells (Fig. 4f, g). Moreover, the rates of apoptotic cells examined by Annexin V staining were significantly increased after treatment with BITC or PEITC (Fig. 4h and Supplementary Fig. S11) in a dose-dependent manner (Supplementary Fig. S12). These results overall demonstrate that BITC and PEITC can trigger the cleavage and mitochondrial translocation of BID and strongly promote apoptosis. In line with these results, BITC and PEITC inhibited cell cycle progression in HeLa cells at the G2/M phase (Supplementary Fig. S13) and suppressed cell proliferation more potently than SFN (Supplementary Fig. S14). Notably, knockout of BID using siRNA attenuated the cytotoxicity of BITC and PEITC, but only weakly affected that of SFN (Supplementary Fig. S15), implying an important role of BID in mediating the cytotoxicity of BITC and PEITC.

### PEITC inhibits the interaction between *N*- and *C*-terminal regions of BID

**Fig. 5:**
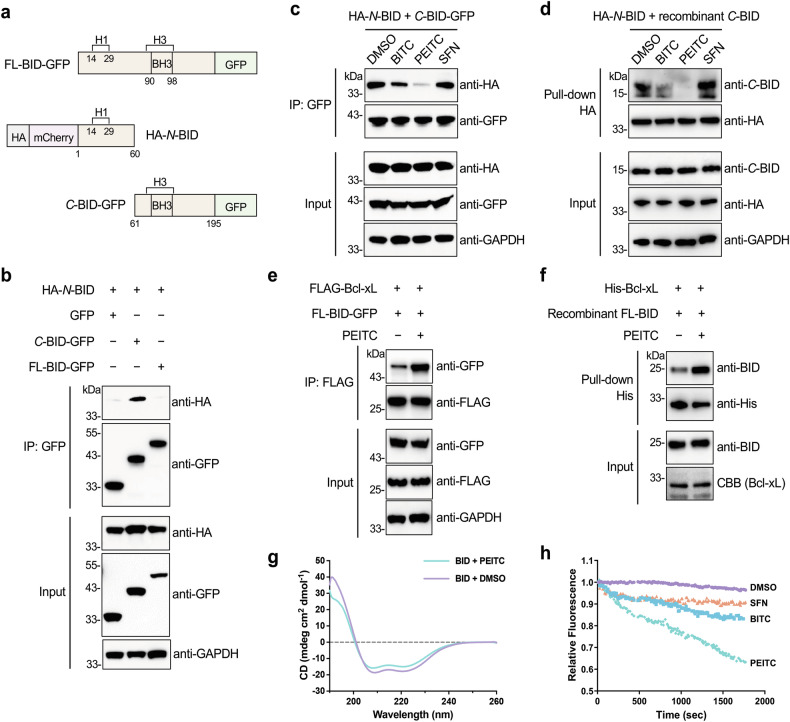
PEITC impairs the *N*- and *C*-terminal interaction of BID. **a** Schematic of BID constructs used in this study. **b** Co-immunoprecipitation analysis of the interaction between *N*-BID and *C*-BID. Lysates from HEK293T cells expressing the indicated proteins were subjected to anti-GFP immunoprecipitation and Western blotting detection of co-immunoprecipitated HA-tagged *N*-BID. **c** Co-immunoprecipitation analysis of the effects of ITCs on the interaction between *N*-BID and *C*-BID. HEK293T cells expressing the indicated proteins were treated with ITCs (20 µM) for 4 h and lysed for anti-GFP immunoprecipitation and Western blotting detection of co-immunoprecipitated HA-tagged *N*-BID. **d** In vitro pull-down analysis of the effects of ITCs on the association between *N*-BID and *C*-BID. Lysates from HEK293T cells expressing HA-tagged *N*-BID were treated with ITCs (20 µM) for 1 h, incubated with recombinant *C*-BID for 1 h, and subjected to anti-HA pull-down and Western blotting analysis with an anti-BID antibody. **e** Co-immunoprecipitation analysis of the effects of PEITC on the interaction between Bcl-xL and BID. HEK293T cells expressing the indicated proteins were treated with PEITC (20 µM) for 4 h and lysed for anti-FLAG immunoprecipitation and Western blotting detection of co-immunoprecipitated GFP-tagged BID. **f** In vitro pull-down analysis of the effects of PEITC on the association between Bcl-xL and BID. Recombinant BID protein was treated with PEITC (20 µM) for 0.5 h, incubated with recombinant His-tagged Bcl-xL for 0.5 h, and subjected to His-tag pull-down and Western blotting analysis with an anti-BID antibody. **g** Secondary structural changes of BID upon binding with PEITC. CD spectra of recombinant BID (5 µM) before and after the treatment with PEITC (20 µM) were recorded. **h** Tertiary structural changes of BID upon binding with ITCs. Changes in BID intrinsic tryptophan fluorescence induced by ITCs (20 µM) were monitored with time.

BID is cleaved by caspase-8 at the D60 residue into *N*- and *C*-terminal fragments [49], referred to as *N*-BID and *C*-BID, respectively. Previous studies have shown that the *N*-BID segment is associated with the *C*-BID segment in both full-length and caspase-8-cleaved BID to serve as an internal inhibitor of its pro-apoptotic activity [36, 50–52]. We thus asked whether covalent modifications of *N*-terminal BID by ITCs affected the interaction between *N*-BID and *C*-BID. To examine this interaction, we established a co-immunoprecipitation assay that utilized co-expression of *N*-BID (residues 1–60) bearing an *N*-terminal HA tag and *C*-BID (residues 61–195) containing a *C*-terminal GFP tag (Fig. 5a) in HEK293T cells [51]. The lysates were subjected to anti-GFP co-immunoprecipitation and anti-HA western blotting. We confirmed the specific interaction between *N*-BID and *C*-BID that was not affected by protein tags (Fig. 5b and Supplementary Fig. S16). *N*-BID was readily co-immunoprecipitated with *C*-BID, but not with full-length BID (FL-BID) due to the *N*-terminal region of FL-BID competing with *N*-BID for the interaction with the *C*-terminal region of FL-BID [51] (Fig. 5b), further verifying the specificity of our co-immunoprecipitation assay. Strikingly, PEITC significantly attenuated the co-immunoprecipitation between *N*-BID and *C*-BID, which was only slightly disturbed by BITC and hardly affected by SFN (Fig. 5c). The in vitro pull-down assay using recombinantly purified *C*-BID and cellularly expressed *N*-BID also demonstrated that the association between *N*-BID and *C*-BID was completely suppressed by PEITC and slightly diminished by BITC, but not affected by SFN (Fig. 5d). Therefore, these data suggest that ITCs can inhibit the interaction between *N*- and *C*-terminal fragments of BID, with the relative efficiency of PEITC > BITC > SFN.

The *C*-terminal fragment of BID also binds with the anti-apoptotic protein Bcl-xL to exert its apoptotic effect [36]. Our co-immunoprecipitation experiments confirmed that *C*-BID was indeed strongly associated with Bcl-xL (Supplementary Fig. S17). FL-BID only weakly interacted with Bcl-xL, because the *N*-terminal region of FL-BID intramolecularly interacted with and masked the *C*-terminal region of FL-BID from interacting with Bcl-xL. Notably, the co-immunoprecipitation of Bcl-xL with FL-BID was significantly increased upon PEITC treatment (Fig. 5e). Similarly, the in vitro pull-down assay showed that PEITC enhanced the pull-down efficiency of recombinant FL-BID by Bcl-xL (Fig. 5f). Overall, these results strongly support that PEITC disrupts the intramolecular interaction between *N*- and *C*-terminal regions of BID, resulting in exposure of the *C*-terminal region for binding with Bcl-xL.

### PEITC alters BID secondary and tertiary structures

To understand the disruptive effects of PEITC on the intramolecular interaction in FL-BID, we evaluated whether PEITC could cause conformational changes in BID. We employed circular dichroism (CD) spectroscopy to detect secondary structural changes of BID [53]. The CD spectrum of recombinant BID exhibited two minima at 208 and 222 nm and a maximum at 192 nm (Fig. 5g). After treatment with PEITC, the ellipticity at both 208 and 222 nm was reduced (Fig. 5g), suggesting a decrease of the α-helical content (from 65.4% to 40.3%) and secondary structural changes of BID. To investigate changes in BID tertiary structure, the intrinsic tryptophan fluorescence was monitored [54]. Recombinant BID, containing only one tryptophan W51 in a large unstructured loop, showed a significant decay of tryptophan fluorescence in a time-dependent manner after the addition of PEITC (Fig. 5h), indicating tertiary structural changes of BID. By contrast, tryptophan fluorescence of BID remained almost constant in the absence of ITCs and decreased to a smaller extent following the addition of BITC or SFN (Fig. 5h). Overall, these results suggest that PEITC modifications result in BID conformational changes, which explains the effects of PEITC in impairing the *N*- and *C*-terminal interaction of BID.

### *N*-terminal cysteines are critical for the interaction between *N*- and *C*-terminal regions of BID

**Fig. 6:**
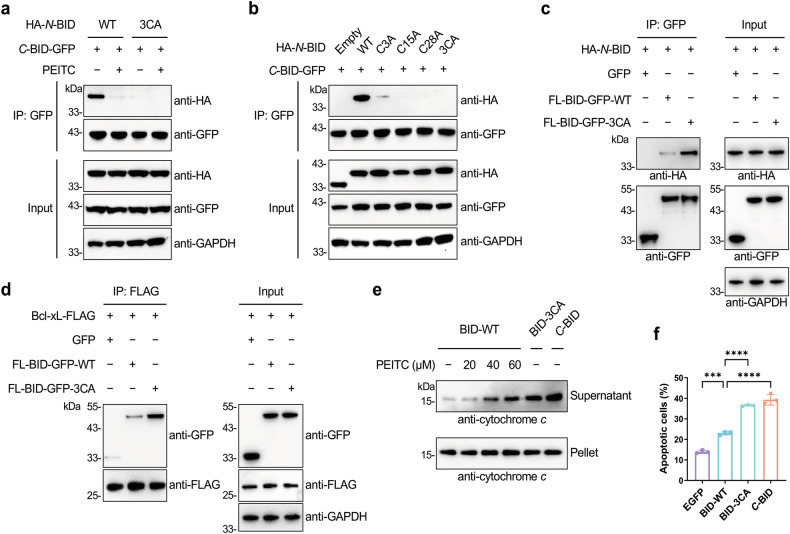
*N*-terminal cysteines are required for the *N*- and *C*-terminal interaction of BID. **a** Co-immunoprecipitation analysis of the effects of PEITC and cysteine mutations on the interaction between *N*-BID and *C*-BID. HEK293T cells expressing the indicated proteins were treated with PEITC (20 µM) for 4 h and lysed for anti-GFP immunoprecipitation and Western blotting detection of co-immunoprecipitated HA-tagged *N*-BID. **b** Co-immunoprecipitation analysis of the effects of cysteine mutations on the interaction between *N*-BID and *C*-BID. HEK293T cells expressing the indicated proteins were lysed for anti-GFP immunoprecipitation and Western blotting detection of co-immunoprecipitated HA-tagged *N*-BID. **c** Co-immunoprecipitation analysis of the effects of cysteine mutations on the interaction between *N*-BID and FL-BID. HEK293T cells expressing the indicated proteins were lysed for anti-GFP immunoprecipitation and Western blotting detection of co-immunoprecipitated HA-tagged *N*-BID. **d** Co-immunoprecipitation analysis of the effects of cysteine mutations on the interaction between Bcl-xL and FL-BID. HEK293T cells expressing the indicated proteins were lysed for anti-FLAG immunoprecipitation and Western blotting detection of co-immunoprecipitated GFP-tagged FL-BID. **e** Western blotting analysis of cytochrome *c* release from mitochondria induced by BID proteins. Recombinant BID proteins were cleaved by caspase-8, treated with or without PEITC at indicated concentrations, incubated with purified mitochondria, and centrifuged. The pellet and supernatant were analyzed for cytochrome *c* release. **f** *N*-terminal cysteine mutations enhance the apoptotic activity of BID. HeLa cells were transfected to express the indicated proteins and assayed for apoptotic cells using Annexin V staining and flow cytometry. Data are shown as mean ± sd (*n* = 3). Statistical analysis was performed with one-way ANOVA test (***p < 0.001, ****p < 0.0001).

The above results demonstrate that PEITC covalently modifies *N*-terminal cysteines of BID and impairs the interaction between *N*- and *C*-terminal regions. We thus asked whether the *N*- and *C*-terminal interaction was directly affected by PEITC modifications at the *N*-terminus. Co-immunoprecipitation experiments using *C*-BID and *N*-BID variants were performed. Surprisingly, the *N*-BID-3CA mutant, devoid of cysteines and unmodifiable by ITCs, did not co-immunoprecipitate with *C*-BID (Fig. 6a), whereas PEITC was repeatedly shown to block the association of *N*-BID with *C*-BID more efficiently than BITC and SFN (Fig. 6a and Supplementary Fig. S18). Further co-immunoprecipitation experiments using additional *N*-BID mutants demonstrated that *N*-terminal cysteines, especially C15 and C28, were required for the interaction of *N*-BID with *C*-BID (Fig. 6b). Consistently, both co-immunoprecipitation and pull-down assays showed that the FL-BID-3CA mutant was associated with *N*-BID and Bcl-xL more avidly than WT FL-BID (Fig. 5c, d and Supplementary Fig. S19), attributed to that the intramolecular interaction between *N*- and *C*-terminal regions of BID was disrupted by cysteine mutations to unmask the *C*-terminal region for interacting with *N*-BID and Bcl-xL. In support of this, CD spectroscopy showed that the ellipticity and α-helical content of recombinant FL-BID-3CA were decreased relative to WT FL-BID (Supplementary Fig. S20), suggesting an altered conformation of BID upon cysteine mutation.

Because the *N*- and *C*-terminal interaction of BID has an inhibitory effect on its pro-apoptotic activity, we envisioned that disruption of this interaction by PEITC modification or cysteine mutagenesis could relieve the self-inhibition and enhance the apoptotic activity of BID. We thus evaluated cytochrome *c* release from isolated mitochondria using recombinant BID proteins. Caspase-8-cleaved WT BID was almost incapable of inducing cytochrome *c* release, whereas *C*-BID was highly active in this regard (Fig. 6e). An increasing amount of cytochrome *c* was released from mitochondria when the caspase-8-cleaved BID complex was treated with PEITC at increasing concentrations. More importantly, caspase-8-cleaved BID-3CA was shown to be as active as *C*-BID in triggering cytochrome *c* release (Fig. 6e). In consistency with these in vitro results, the Annexin V staining assay demonstrated that *C*-BID and FL-BID-3CA, bearing a disrupted intramolecular interaction, were similarly active and both much more potent than WT FL-BID in inducing apoptosis (Fig. 6f). These data overall suggest that *N*-terminal cysteines are essential for regulating the intramolecular interaction and apoptotic activity of BID.

## Discussion

**Fig. 7:**
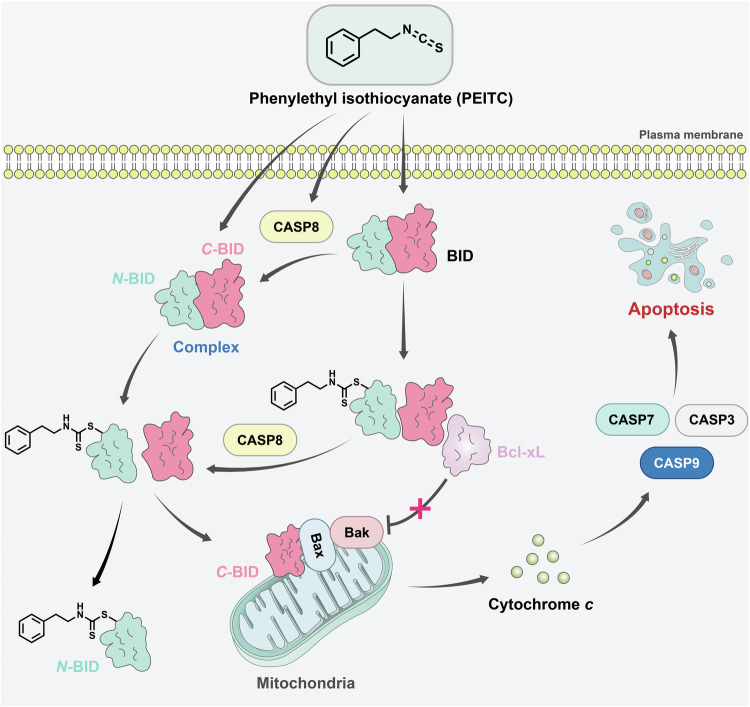
A working model illustrating the mechanism by which PEITC promotes apoptosis. PEITC covalently modifies the *N*-terminal cysteines of BID, disrupting the interaction between the *N*- and *C*-terminal fragments of BID. This releases the pro-apoptotic *C*-terminal fragment from the autoinhibitory noncovalent complex to induce cytochrome *c* release and promote apoptosis. Simultaneously, PEITC modification exposes the BH3 domain in the *C*-terminal region, enabling BID to bind with Bcl-xL to suppress its anti-apoptotic activity.

In this study, we developed alkynyl-functionalized ITC-derived chemical probes for globally profiling the target proteins of ITCs, identified BID as a novel target of BITC, PEITC, and SFN in human cancer cells, and revealed the function of covalent binding of BID with PEITC in mediating apoptosis. Mechanistically, as an early event following treatment, PEITC promiscuously modifies the *N*-terminal cysteines of full-length BID to form dithiocarbamate adducts. This modification induces conformational changes in BID, disrupting the autoinhibitory intramolecular interaction within full-length BID and exposing the BH3 domain in the *C*-terminal region. As a result, this exposure allows BID to bind anti-apoptotic proteins such as Bcl-xL, thereby suppressing their anti-apoptotic activity. Meanwhile, caspase-8 is activated shortly after PEITC treatment [12], leading to the cleavage of PEITC-modified full-length BID. On the other hand, caspase-8 may cleave unmodified full-length BID first, forming a noncovalent complex between the *N*- and *C*-terminal fragments, followed by the covalent modification by PEITC. In both scenarios, the PEITC modification disrupts the noncovalent complex between *N*- and *C*-terminal fragments of BID. Thus, the highly pro-apoptotic *C*-terminal fragment is released from the autoinhibitory complex and translocated to the mitochondrial outer membrane to induce cytochrome *c* release [55], which further activates the caspase cascade and ultimately leads to apoptotic cell death (Fig. 7). Our findings highlight that the covalent interaction between PEITC and an apoptosis regulatory protein, i.e., BID, can be an important initiating event for inducing apoptosis.

Although previous studies have implicated that binding of ITCs to intracellular proteins may trigger apoptosis, the direct targets of ITCs underlying apoptosis induction remained unclear for decades [24]. Importantly, we have for the first time discovered the covalent binding of BID with PEITC and revealed its functional role in relieving the self-inhibitory interaction within BID to promote apoptosis. We found that the labeling efficiency of BITC-yne, PEITC-yne, and SFN-yne largely correlates with the ability of BITC, PEITC, and SFN to induce BID conformational changes, which is further in high accordance with their capability to disrupt the *N*- and *C*-terminal interaction, as well as the potency to induce BID cleavage and apoptosis in the order of PEITC > BITC > SFN. Therefore, the affinities of ITCs for covalently binding to BID and the functions of these modifications greatly depend on the exact structures of ITCs, although they all contain the isothiocyanate functionality.

Recent large-scale chemical proteomics profiling has identified thousands of functional and potentially druggable cysteines in complexed proteomes [56–58]. However, BID cysteines were only identified in a few of these studies [59] and potential ligands targeting BID cysteines were still unavailable. Notably, our study discovered the first small molecule that covalently modifies BID cysteines to unleash its pro-apoptotic activity, highlighting that BID can be druggable with the development of more effective and selective ITC-related compounds for the induction of apoptosis.

BID contains eight α-helices, including helix H1 (14–29) in the *N*-terminal region and helix H3 containing the BH3 domain (Fig. 5a) [36, 37]. The solution structure of human BID implicates that helix H1 has close contacts with helix H3 and may negatively regulate the apoptotic function of BID [36]. However, the precise autoinhibitory function conferred by helix H1 remains unexplored. We demonstrate that cysteines (C15 and C28) located in helix H1 are required to maintain the intramolecular interaction with the *C*-terminus, thus experimentally defining the essential role of helix H1 in regulating the pro-apoptotic potential of BID.

In summary, this work provides important insights into the long-standing question of how ITC binding to intracellular proteins directly contributes to the induction of apoptosis. Our findings also demonstrate that the pro-apoptotic potential of BID is negatively regulated by *N*-terminal cysteines, indicating that these cysteines could be targeted by covalent inhibitors for the induction of apoptosis and potential cancer therapeutics.

## Materials and methods

### Reagents and antibodies

BITC, PEITC, SFN, IAA, NEM, and other reagents were purchased from Sigma and MedChemExpress unless otherwise noted. Azido-rhodamine and azido-biotin were synthesized according to the literature [60]. EDTA-free protease inhibitor cocktail was purchased from Roche. Q5 High-Fidelity DNA polymerase, restriction enzymes, and dNTPs were obtained from New England Biolabs. Oligonucleotide primers were synthesized by Tsingke. Plasmid DNA was purified with Plasmid Mini Kit (Omega). Polyethylenimine (PEI) was purchased from Polysciences. Viafect was purchased from Promega. Sequencing-grade trypsin was obtained from Promega. Anti-BID (2794, 1:1000 dilution) and anti-PARP1 (9532, 1:2000 dilution) were purchased from Cell Signaling Technology. Anti-caspase-3 (19677-1-AP, 1:1000 dilution), anti-caspase-8 (13423-1-AP, 1:1000 dilution), anti-caspase-9 (10380-1-AP, 1:1000 dilution), anti-6xHis-HRP (HRP-66005, 1:5000 dilution), anti-GFP (66002-1-Ig, 1:10000 dilution), anti-cytochrome C (10993-1-AP, 1:2000 dilution), and anti-GAPDH-HRP (HRP-60004, 1:10000 dilution) were purchased from Proteintech. Anti-HA-HRP (3F10, 1:1000 dilution) was purchased from Roche. Anti-Flag M2 antibody (F1804, 1:1000) was purchased from Sigma. Goat anti-mouse-HRP and anti-rabbit-HRP secondary antibodies (1:10000 dilution) were purchased from Jackson ImmunoResearch Laboratories. siRNAs were purchased from Genepharma. The siRNA sequences for BID are as follows: GGAGGAGCACAGUGCGGAUTT (siRNA-BID-1), GCCUCAGGGAUGAGUGCAUTT (siRNA-BID-2), GCCGUCCUUGCUCCGUGAUTT (siRNA-BID-3), GCACCUACGUGAGGAGCUUTT (siRNA-BID-4).

### Plasmid construction

cDNAs encoding proteins of interest were purchased from MiaoLing Plasmid Platform. Human BID was cloned into pCMV-C-HA vector with a *C*-terminal HA tag or cloned into pEGFP-C1 vector with a *C*-terminal GFP tag. *N*-terminal fragment of BID (residues 1–60) was cloned into pmCherry vector with an *N*-terminal HA tag followed by a mCherry tag. *C*-terminal fragment of BID (residues 61–195) was cloned into pEGFP-C1 vector with a *C*-terminal GFP tag. Mouse BID, MIF, and DDT were cloned into pCMV-3xHA vector with a *C*-terminal 3xHA tag. Bcl-xL was cloned into pEnCMV vector with a *C*-terminal 3xFlag tag. KEAP1 and MAP4 were cloned into pCMV-N-HA vector with an *N*-terminal HA tag. TXN and LSM14A were cloned into pCMV-C-HA vector with a *C*-terminal HA tag. For bacterial expression, BID, Bcl-xL, and caspase-8 were cloned into pET28a vector with an *N*-terminal 6xHis tag. Site-directed mutagenesis was performed with QuikChange II Site-Directed Mutagenesis Kit (Agilent). Primers were designed with QuikChange Primer Design Program. All plasmids were verified by sequencing.

### Cell culture and transfection

HEK293T, HeLa, and MCF-7 cells were obtained from the American Type Culture Collection (ATCC). All cells were authenticated by STR profiling and confirmed to be free of mycoplasma contamination. Cells were grown in Dulbecco’s modified Eagle’s medium (DMEM; Cytiva) supplemented with 10% fetal bovine serum (FBS; Sigma-Aldrich) and cultured at 37 °C in a 5% CO_2_ humidified incubator. For transfection, cells were grown to 70–80% confluence and transfected with indicated plasmids using PEI ( ~ 2.5:1 ratio of PEI/DNA) or Viafect (~3:1 ratio of Viafect/DNA) for 18–24 h in culture media. For siRNA transfection, HeLa cells were transfected with the siRNA using siRNA-Mate plus transfection reagent (Genepharma; G04026) for 48 h.

### CuAAC click reaction and in-gel fluorescence analysis

Cells were seeded in 12-well plates under standard cell culture conditions and incubated with ITC probes in DMEM at 37 °C for 30 min unless otherwise noted. For competition experiments, cells were pre-treated with the competitor for 30 min and then incubated with the ITC probe in DMEM at 37 °C for another 30 min. Cells were harvested, washed three times with PBS, and lysed with 1% SDS lysis buffer (1% SDS, 150 mM NaCl, 50 mM HEPES, pH 7.4, supplemented with benzonase) at room temperature with vigorous vortexing. Cell lysates were centrifuged at 12,000 × *g* for 20 min and quantified using the BCA assay (Pierce). Cell lysates (100 µg) were normalized with lysis buffer to an equal protein concentration (89 µL) and reacted with 11 µL freshly prepared click master mix containing 100 µM azido-rhodamine, 1 mM tris-(2-carboxyethyl) phosphine hydrochloride (TCEP), 100 µM tris-[(1-benzyl-1H-1,2,3-triazol-4-yl)methyl]amine (TBTA), and 1 mM CuSO_4_ at room temperature in the dark for 2 h. The reactions were quenched by methanol and placed at −20 °C overnight. The proteins were precipitated by centrifugation at 20,000 × *g* at 4 °C for 20 min, washed with methanol, and air-dried. The resulting protein pellets were resuspended with 1× reducing SDS-loading buffer, heated at 95 °C for 5 min, and loaded onto 4–20% ExpressPlus gels (Genscript) for SDS-PAGE separation. For in-gel fluorescence, gels were scanned on a ChemiDoc MP Imager (Bio-Rad) with the rhodamine filter and stained with Coomassie Brilliant Blue (CBB) as loading control.

### Competition-based quantitative chemical proteomics

Quantitative chemical proteomics experiments were performed according to our previous report [44]. Briefly, MCF-7 cells were cultured in arginine and lysine-deficient DMEM (ThermoFisher) supplemented with 10% dialyzed FBS (ThermoFisher). SILAC media containing ^13^C_6_,^15^N_2_-L-lysine-2HCl (Lys8; Cambridge Isotope) and ^13^C_6_,^15^N_4_-L-arginine-HCl (Arg10; Cambridge Isotope) were used to culture heavy-labeled cells, whereas SILAC media containing L-lysine-2HCl (Lys0; Sigma) and L-arginine-HCl (Arg0; Sigma) were used to culture light-labeled cells. After seven cell doublings, the incorporation of heavy isotopes was examined by LC-MS/MS analysis.

For the forward SILAC analysis, heavy-labeled cells were treated with the ITC probe (20 µM) for 30 min and light-labeled cells were pre-treated with the natural ITC (60 µM) for 30 min, followed by the ITC probe (20 µM) for another 30 min. For the reverse SILAC analysis, light-labeled cells were treated with the ITC probe (20 µM) for 30 min and heavy-labeled cells were treated with the natural ITC (60 µM) for 30 min, followed by the ITC probe (20 µM) for another 30 min. Cells were harvested and lysed with 1% SDS lysis buffer (containing Roche protease inhibitor cocktail and benzonase). Cell lysates were centrifuged at 13,000 × *g* for 15 min and quantified using the BCA assay (Pierce). Heavy- and light-labeled cell lysates were mixed equally and clicked with azido-biotin as described above. Proteins were precipitated and washed with methanol. The air-dried protein pellets were resuspended with 1% SDS buffer and incubated with prewashed streptavidin agarose beads (ThermoFisher) on a nutating mixer at room temperature for 2 h. The beads were washed six times with 1% SDS in PBS, 5 M urea in PBS, PBS, and ammonium bicarbonate (ABC) buffer (100 mM) successively. The beads were reduced with TCEP (10 mM) for 30 min, alkylated by IAA (20 mM) for 30 min in the dark, washed with ABC buffer, and digested with trypsin (0.5 µg) at 37 °C overnight. The supernatants were collected, dried, and fractionated for LC-MS/MS analysis.

Samples were analyzed with a Q Exactive HF-X Hybrid Quadrupole-Orbitrap mass spectrometer (ThermoFisher) coupled with an EASY-nLC 1200 system (ThermoFisher) using buffer A (water with 0.1% formic acid) and buffer B (80% acetonitrile in water with 0.1% formic acid) with a flow rate of 0.3 µL/min for elution. Peptides were loaded onto an Acclaim PepMap C18 reverse-phase column (ThermoFisher). The Q Exactive HF-X mass spectrometer was calibrated using the Tune software and configured for the data-dependent acquisition mode using a full MS/DD–MS/MS setup. Data were acquired in profile mode with positive polarity over the mass range from 350 to 1800 with an MS resolution of 60,000 at m/z 200. Other parameters are as follows: automatic gain control (AGC) target, 3 × 10^6^; maximum ion injection (IT) time, 50 ms; MS2 resolution, 15,000 at m/z 200; intensity threshold, 2.2 × 10^4^; isolation width, 1.6 m/z; normalized collision energy, 28%; dynamic exclusion, 30 s.

LC-MS/MS data were analyzed with MaxQuant [61] v1.5.3.8 and searched against the Human UniProt Reference Proteome database (UP000005640, modified on January 5, 2021; 20609 entries, one protein sequence per gene without isoforms) with known contaminants. Multiplicity was set to 2 with heavy labels of Lys8 and Arg10. Maximal two missed cleavage were allowed. Cysteine carbamidomethylation was set as a fixed modification. Methionine oxidation and *N*-terminal acetylation were included as variable modifications. The first search peptide tolerance and allowed fragment mass deviation were set to 20 ppm. The false discovery rates for peptide spectrum match were set to 1%. Only unique and razor peptides were used for quantification with minimal two ratio counts. The re-quantify feature of MaxQuant was enabled. Other parameters in MaxQuant were used as default. The search results were analyzed with Excel. Known contaminants, reverse hits, and hits identified only by site were removed. Normalized SILAC ratios were transformed with log2.

### Immunoprecipitation and in-gel fluorescence analysis of candidate proteins

HEK293T cells were transfected with indicated plasmids to express HA-tagged candidate proteins for 16–24 h and labeled with ITC probes in DMEM for 30 min. Competition experiments were performed as described above. Cells were harvested and lysed with chilled 1% Triton X-100 lysis buffer with vortexing at 4 °C. Cell lysates were centrifuged at 12,000 × *g* for 20 min at 4 °C and quantified with the BCA assay (Pierce). Anti-HA agarose beads (ThermoFisher) were incubated with lysates ( ~ 1 mg) on a rotator at 4 °C for 2 h and washed three times with RIPA buffer and PBS. The beads were resuspended in PBS and reacted with freshly prepared click master mix as described above at room temperature in the dark for 2 h. The beads were washed three times with RIPA buffer, resuspended with 1× reducing SDS-loading buffer, and heated for 5 min at 95 °C. The supernatants were loaded onto 4–20% gels for SDS-PAGE separation and in-gel fluorescence analysis. Another gel loaded with identical samples was processed for Western blotting analysis.

### Cell viability assay

MTT (3-(4,5-Dimethylthiazol-2-yl)-2,5-diphenyltetrazolium bromide) assay was used to examine the cytotoxicity. Cells were seeded in 96-well plates and treated with natural ITCs or ITC probes at different concentrations for 72 h. MTT solution (Beyotime, C0009; 5 mg/mL in PBS) was added into each well (10 µL) and incubated with cells at 37 °C for 4 h. Formazan solubilization solution was added into each well (100 µL) and incubated with cells at 37 °C until purple formazan crystals were completely dissolved. The absorbance was measured at 570 nm with a Cytation 5 Multi-Mode Reader (BioTek). GI_50_ values were calculated using the GraphPad Prism software.

### Cell cycle analysis

Cells were seeded in 24-well plates and treated with ITC probes for 72 h. Cells were detached with trypsin, harvested, and washed with cold PBS twice. The cell pellets were resuspended in cold 75% EtOH/H_2_O solution with slow vortexing and fixed at −20 °C overnight. The cell suspensions were centrifuged at 1,500 × *g* and washed with PBS twice. Cells were resuspended in propidium iodide (PI) staining buffer (PBS containing 100 µg/mL RNase A and 50 µg/mL PI) at room temperature in the dark for 30 min. Cells were analyzed on an Attune NxT flow cytometer (ThermoFisher) with three replicates. PI fluorescence was collected at 585±16 nm with excitation at 561 nm.

### Caspase-3/7 activity assay

Caspase-3/7 activity was measured with the caspase-3/7 assay kit (Beyotime, C1168S). Briefly, cells were treated with natural ITCs or ITC probes for 4 h, harvested with trypsin, and washed with cold PBS. Cell pellets were resuspended in 200 µL PBS containing 5 µM GreenNuc caspase-3 substrate (Beyotime) and incubated at room temperature in the dark for 20 min. Cells were analyzed on an Attune NxT flow cytometer (ThermoFisher) with three replicates. Fluorescence was collected at 530 ± 30 nm with excitation at 488 nm.

### Annexin V apoptosis assay

Apoptotic cells were measured with the Annexin V-FITC apoptosis detection kit (Beyotime, C1062S). Briefly, cells were treated with natural ITCs or ITC probes for 4 h, harvested with trypsin, and washed with cold PBS. Cell pellets were resuspended in Annexin V-FITC binding buffer (containing Annexin V-FITC and PI) and incubated at room temperature in the dark for 20 min. Cells were analyzed on an Attune NxT flow cytometer (ThermoFisher) with three replicates. FITC fluorescence was collected at 530 ± 30 nm with excitation at 488 nm.

### Mitochondrial membrane potential assay

Mitochondrial membrane potential was measured with the mitochondrial membrane potential assay kit with TMRE (Beyotime, C2001S). Briefly, cells were treated with natural ITCs or ITC probes for 4 h, incubated with TMRE and Hoechst 333422 in the assay buffer at 37 °C for 30 min. Cells were washed with PBS and imaged in FluoroBrite DMEM (ThermoFisher) with a Nikon A1R confocal fluorescence microscope. Hoechst 333422 was excited at 405 nm and emission was collected from 425 to 475 nm. TMRE was excited at 561 nm and emission was collected from 570 to 620 nm. For quantification analysis, the fluorescence intensity of each cell was quantified with ImageJ (NIH). For each condition, multiple cells (reported as *n*) in replicate wells were analyzed.

### Fluorescence imaging of BID

HeLa cells were co-transfected with plasmids encoding TOMM-mCherry and FL-BID-GFP, *C*-BID-GFP (AA 61–195), or the EGFP-C1 vector for 16 h and treated with natural ITCs for 4 h. After that, cells were stained with Hoechst 333422 at 37 °C for 30 min, washed with PBS, and imaged in FluoroBrite DMEM (ThermoFisher) with a Nikon A1R confocal fluorescence microscope. GFP was excited at 488 nm and emission was collected from 500 to 550 nm. mCherry was excited at 561 nm and emission was collected from 570 to 620 nm.

### Co-Immunoprecipitation

HEK293T cells were co-transfected to express HA-*N*-BID and *C*-BID-GFP for 16 h and treated with natural ITCs for 4 h. Cells were harvested, washed with PBS, and lysed with chilled 1% Triton buffer containing Roche protease inhibitor cocktail at 4 °C. Cell lysates were centrifuged at 12,000 × *g* at 4 °C for 20 min and quantified with the BCA assay (Pierce). Equal amounts of cell lysates (~1 mg) were incubated with anti-GFP nanobody agarose beads (AlpaLifeBio) on a rotator at 4 °C for 2 h. The beads were washed six times with Triton buffer and resuspended with 1× reducing SDS-loading buffer. After heating at 95 °C for 5 min, the supernatants were loaded onto 4–20% gels for SDS-PAGE separation and Western blotting analysis. For co-immunoprecipitation of Bcl-xL with BID, HEK293T cells expressing indicated proteins were used for co-immunoprecipitation with anti-FLAG agarose beads (Sigma) following a similar procedure as described above.

### Recombinant protein purification

Full-length BID proteins (wild-type and mutants) containing an *N*-terminal 6xHis-tag followed by a TEV protease cleavage sequence (ENLYFQS) were expressed in *E. coli* BL21(DE3) (AlpaLife). BID proteins were soluble and purified to homogeneity by standard nickel affinity chromatography (Sangon Biotech). Bcl-xL was similarly expressed and purified by standard nickel affinity chromatography. Active recombinant caspase-8 (residues 207–479) containing a non-cleavable 6xHis-tag at the *N*-terminus was expressed in BL21(DE3)pLysS (AlpaLife) and purified by standard nickel affinity chromatography. Most of the caspase-8 protein was expressed as an insoluble precursor protein. A small fraction of the protein underwent autocatalysis to its active two-subunit form, which was soluble and readily purified. For the preparation of recombinant *C*-BID, FL-BID was incubated with the purified active caspase-8 at a ratio of 100:1 in PIPES buffer (20 mM PIPES, 100 mM NaCl, 10 mM DTT, 1 mM EDTA, 0.1% CHAPS, and 10% sucrose) at room temperature for 24 h. The cleaved *C*-BID was associated with His-tagged *N*-BID, and dissociation of *C*-BID was carried out in 2% n-Octyl-β-D-glucopyranoside with vortexing at room temperature for 30 min. The mixture was subjected to nickel affinity purification and incubated at room temperature for 1 h with vortexing. After centrifugation, the supernatant containing *C*-BID was collected and incubated with nickel affinity resins for 30 min repeatedly. The resulting *C*-BID protein in the supernatant was diluted and dialyzed against PBS.

### In vitro recombinant BID labeling

To label BID proteins in vitro, the recombinant proteins (4 µM) were incubated with ITC probes (20 µM) and TCEP (5 µM) in 50 µL PBS buffer at room temperature in the dark for 2 h. The reactions were quenched by methanol (500 µL) and placed at −20 °C overnight. The proteins were precipitated by centrifugation at 20,000 × *g* at 4 °C for 20 min and air-dried. The pellets were redissolved in 1% SDS buffer and reacted with freshly prepared click master mix at room temperature in the dark for 2 h. The mixtures were diluted with 4× reducing SDS-loading buffer and loaded onto 4–20% gels for SDS-PAGE separation and in-gel fluorescence analysis.

### LC-MS/MS analysis of BID modification sites

The recombinant BID protein (15 µg) was reconstituted in 100 µL ABC buffer and incubated with PEITC (20 µM) in the presence of TCEP (5 µM) at room temperature in the dark for 2 h. Excess PEITC was removed by ultrafiltration. The BID protein was digested with trypsin (0.3 µg) at 37 °C for 2 h. The resulting peptides were analyzed by LC-MS/MS with a Q Exactive HF-X Hybrid Quadrupole-Orbitrap mass spectrometer (ThermoFisher). To identify the modification sites, the LC-MS/MS data were analyzed with pFind [62] and searched against the Human UniProt Reference Proteome database (UP000005640) concatenated with 6xHis-tag BID and known contaminants. Cysteine thiocarbamoylation (+163.04557 Da) was included as a variable modification. Other parameters in pFind were used as default.

### In vitro pull-down assay

For in vitro pull-down of *C*-BID with HA-*N*-BID, HA-*N*-BID was expressed in HEK293T cells through transfection of the HA-*N*-BID plasmid. Cell lysates containing HA-*N*-BID were treated with natural ITCs (20 µM) in the presence of TCEP (5 µM) at 4 °C for 1 h and incubated with recombinant *C*-BID protein (5 µg) for 1 h. The lysates were subjected to anti-HA immunoprecipitation using anti-HA agarose beads on a rotator at 4 °C for 2 h. The beads were washed six times with 1% Triton X-100 buffer, resuspended with 1× reducing SDS-loading buffer, and heated at 95 °C for 5 min. The supernatants were loaded onto 4–20% gels for SDS-PAGE separation and Western blotting analysis. For in vitro pull-down of FL-BID with Bcl-xL, *N*-terminal 6xHis-tag in recombinant BID protein or mutant was removed by TEV protease and incubated with recombinant His-tagged Bcl-xL protein on ice for 0.5 h. The protein mixture was incubated with nickel affinity resins for 2 h and washed with 1% Triton X-100 buffer. Bound proteins were eluted with 1× reducing SDS-loading buffer and heated at 95 °C for 5 min. The supernatants were loaded onto 4–20% gels for SDS-PAGE separation and Western blotting analysis.

### In vitro cytochrome c release

Mitochondria were isolated from HEK293T cells using the cell mitochondria isolation kit (Beyotime, C3601). Briefly, cells were harvested and homogenized in mitochondria isolation reagent with a glass homogenizer. The homogenates were centrifuged at 600 × *g* at 4 °C for 10 min. The supernatants were collected and further centrifuged at 11,000 × *g* at 4 °C for 10 min to pellet the mitochondria. BID recombinant proteins were cleaved by recombinant caspase-8 at 4 °C for 12 h. The resulting cleaved BID complexes were reconstituted in mitochondrial storage buffer and treated with ITCs in the presence of TCEP at room temperature for 30 min. The ITC-treated BID complexes were incubated with isolated mitochondria in mitochondrial storage buffer at 37 °C for 30 min [63]. After that, mitochondria were pelleted by centrifugation at 11,000 × *g* at 4 °C for 10 min. The supernatants were collected and diluted with 4× reducing SDS-loading buffer. The pelleted mitochondria were solubilized with 1× reducing SDS-loading buffer in a volume equal to that of the supernatant. Samples were heated at 95 °C for 5 min and analyzed by Western blotting.

### Circular dichroism

Circular dichroism spectra of recombinant BID proteins (5 µM) in PBS buffer (pH 6.8) before and after the addition of ITCs (20 µM) were recorded on a Chirascan circular dichroism spectrometer (Applied Photophysics) over a wavelength range from 190 to 260 nm using a cell of 0.1 cm length. The spectra were averaged over three scans. The helical content was estimated from ellipticity at 190–240 nm using DichroWeb [64].

### Tryptophan fluorescence measurement

Changes of intrinsic tryptophan fluorescence of recombinant BID proteins (5 μM) in PIPES buffer (80 mM PIPES, 1 mM MgCl_2_, 1 mM EGTA, pH 6.8) after addition of ITCs (20 µM) were monitored with time on a Hitachi F-4600 fluorescence spectrophotometer, with emission and excitation wavelengths at 335 nm and 295 nm, respectively.

### Statistical analysis

All experiments were biologically replicated at least three times using different cell cultures in the laboratory, with representative results shown. All data are shown as mean ± sd, calculated from independent biological replicates (*n* = 3 or 6 indicated in the figure legends). The GraphPad Prism software was used to conduct statistical analysis. The method (one-way or two-way ANOVA test without adjustments) for evaluating statistical significance is indicated in the figure legends, with the biological replicate number specified. Significance was denoted as follows: ns = non-significant (*p* > 0.05), *p < 0.05, **p < 0.01, ***p < 0.001, and ****p < 0.0001.

## Supplementary information

Supplementary Information

Supplementary Information

Original Data

## Data Availability

The mass spectrometry proteomics data have been deposited to the ProteomeXchange Consortium via the PRIDE [65] partner repository with the dataset identifier PXD051941. Other data generated in this study are included in this published article. Full and uncropped western blots can be found in Supplementary Information.
