# Supplementary material for: Quantitative chemical proteomics reveals that phenethyl isothiocyanate covalently targets BID to promote apoptosis
Source: Cell Death Discov. 2024 Oct 29;10:456. doi: 10.1038/s41420-024-02225-7 (PMC11522290; doi:10.1038/s41420-024-02225-7)

Supplementary Information

Quantitative chemical proteomics reveal that phenethyl isothiocyanate covalently targets BID to promote apoptosis

Xiaoshu Dong[1#], Xinqian Yu[1#], Minghao Lu[1], Yaxin Xu[1], Liyan Zhou[1], and Tao Peng[1*]

[1] State Key Laboratory of Chemical Oncogenomics, School of Chemical Biology and Biotechnology, Peking University Shenzhen Graduate School, Shenzhen 518055, China

[#] These authors contributed to this work equally.

* Correspondence: Tao Peng, tpeng@pku.edu.cn

Table of Contents

Supplementary Tables 2

Supplementary Figures 2

Chemical Synthesis 17

General methods and materials 17

Synthesis of BITC-yne 17

Synthesis of PEITC-yne 19

Synthesis of SFN-yne 20

References 23

NMR Spectra 24

Supplementary Tables

Table S1. Quantitative chemical proteomics data for profiling the target proteins of ITCs by using ITC-derived chemical probes. (Excel file)

Supplementary Figures

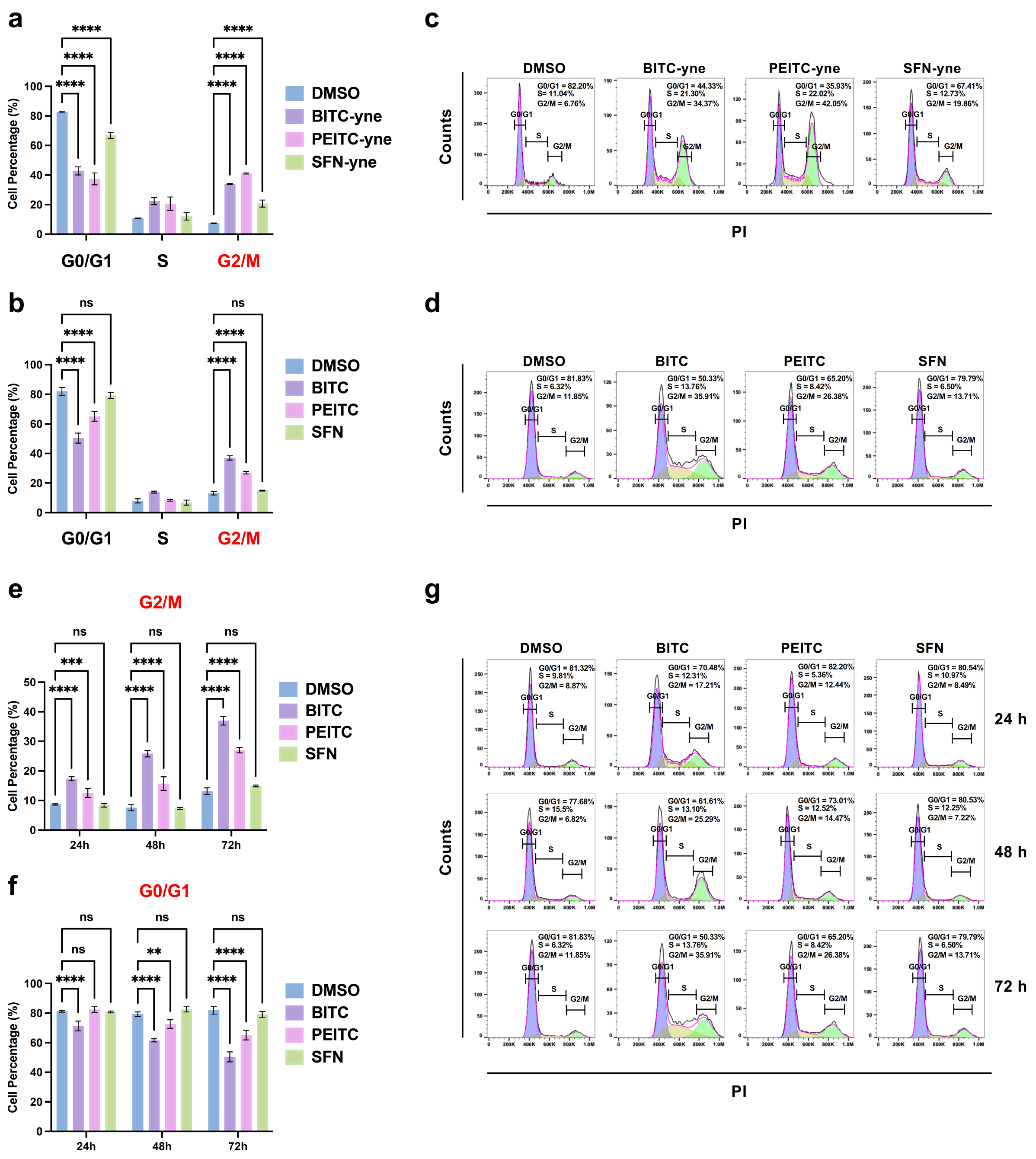

Supplementary Figure S1. ITC probes induce cell cycle arrest at G2/M in MCF-7 cells.

Cells were treated with **a** ITC probes or **b** natural ITCs (2 μM) for 72 h and stained with propidium iodide (PI) for flow cytometry analysis. **c** Representative flow cytometry data of **a** showing that ITC probes induce cell cycle arrest. **d** Representative flow cytometry data of **b** showing that natural ITCs induce cell cycle arrest. **e** and **f** Cells were treated with natural ITCs (2 μM) for different time periods and stained with propidium iodide (PI) for flow cytometry analysis. **g** Representative flow cytometry data of **e** and **f** showing that natural ITCs induce cell cycle arrest. Cell percentages in different phases of cell cycle after treatment with ITC probes or natural ITCs are shown. Data are mean ± sd (*n* = 3). Statistical analysis was performed with two-way ANOVA test (ns $p > 0.05$ non-significant, ∗∗ $p < 0.01$, ∗∗∗ $p < 0.001$, ∗∗∗∗ $p < 0.0001$).

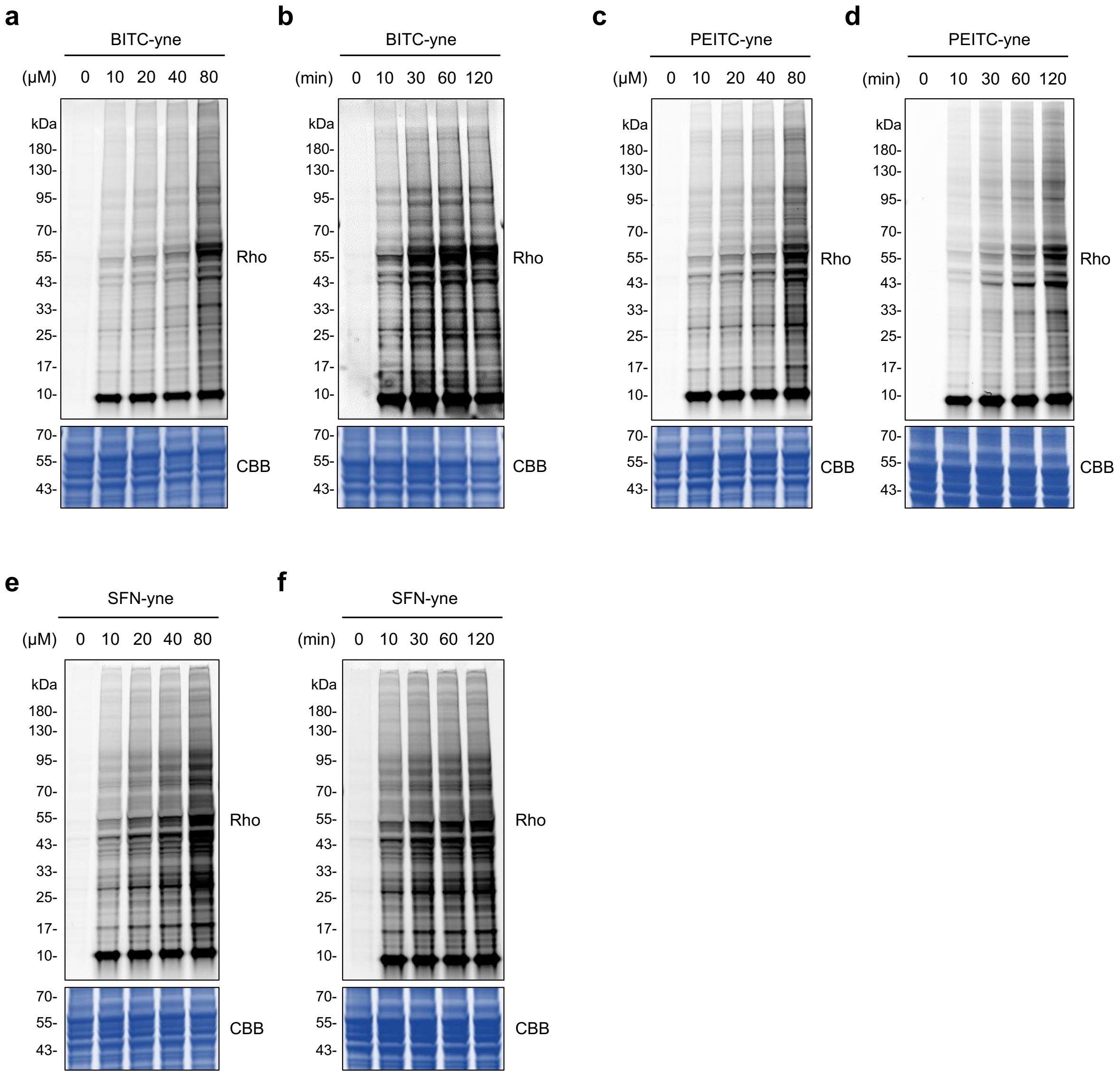

Supplementary Figure S2. Dose- and time-dependent labeling of proteins in live cells by ITC probes. a In-gel fluorescence analysis of dose-dependent labeling of proteins with BITC-yne. **b** In-gel fluorescence analysis of time-dependent labeling of proteins with BITC-yne. **c** In-gel fluorescence analysis of dose-dependent labeling of proteins with PEITC-yne. **d** In-gel fluorescence analysis of time-dependent labeling of proteins with PEITC-yne. **e** In-gel fluorescence analysis of dose-dependent labeling of proteins with SFN-yne. **f** In-gel fluorescence analysis of time-dependent labeling of proteins with SFN-yne. For dose-dependent analyses, MCF-7 cells were labeled with the probe at indicated concentrations for 2 h. For time-dependent analyses, MCF-7 cells were labeled with the probe (20 μM) for indicated periods. Cells were then lysed for conjugation with azido-rhodamine and in-gel fluorescence analysis. Rho: rhodamine; CBB: Coomassie Brilliant Blue staining.

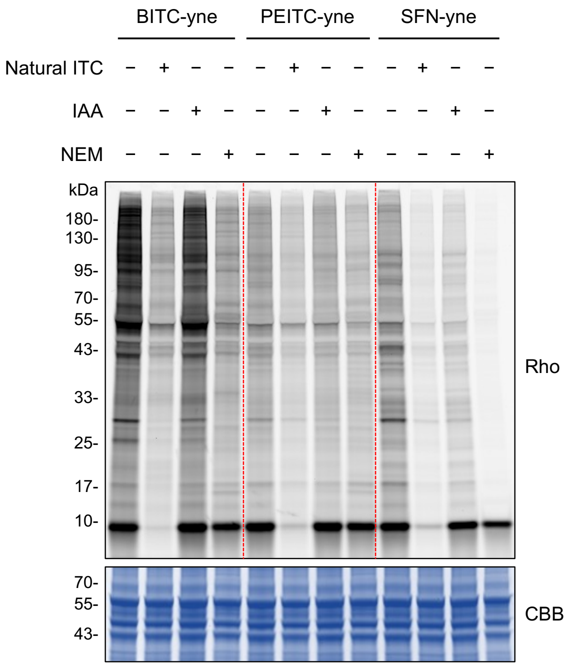

Supplementary Figure S3. Competition of protein labeling by ITC probes in live cells. MCF-7 cells were pre-treated with the corresponding natural ITC (60 μM), iodoacetamide (IAA) (100 μM), or *N*-ethylmaleimide (NEM) (100 μM) for 0.5 h and labeled with the probe (BITC-yne, PEITC-yne, or SFN-yne) (20 μM) for 0.5 h. Cells were then lysed for conjugation with azido-rhodamine and in-gel fluorescence analysis. Rho: rhodamine; CBB: Coomassie Brilliant Blue staining.

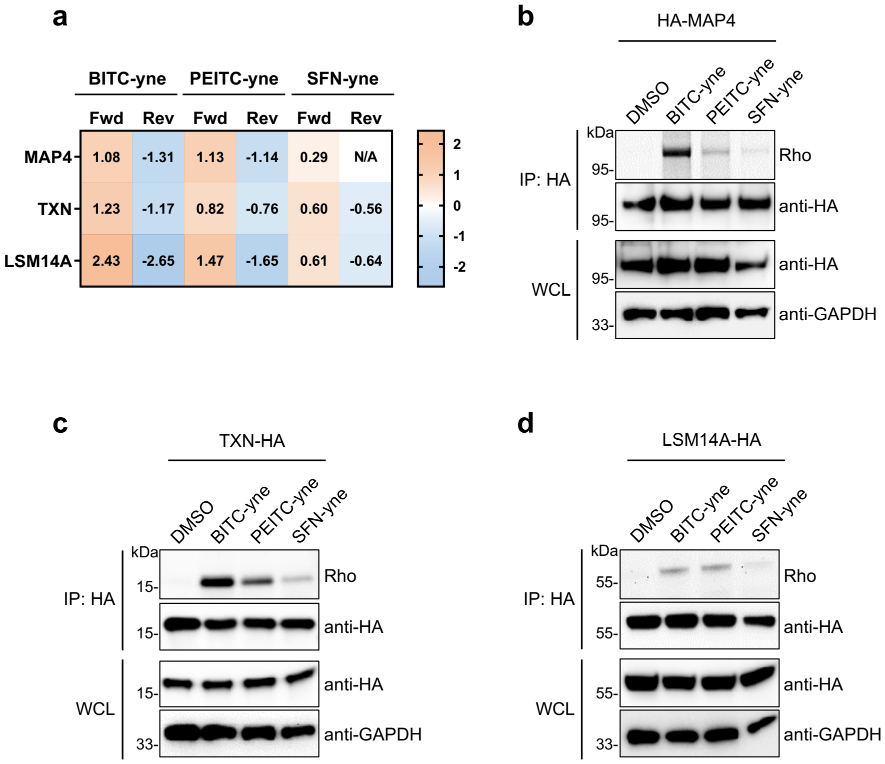

Supplementary Figure S4. Validation of newly identified candidate target proteins of ITCs. a SILAC ratios (with $\log_2$ transformation) of selected candidate target proteins (e.g., MAP4, TXN, and LSM14A) quantified in the quantitative chemical proteomics profiling experiments. **b** In-gel fluorescence validation of MAP4 labeling by ITC probes. **c** In-gel fluorescence validation of TXN labeling by ITC probes. **d** In-gel fluorescence validation of LSM14A labeling by ITC probes. HEK293T cells were transfected to express the candidate target protein, labeled with the probe (20 μM) for 0.5 h, and subjected to immunoprecipitation (IP), followed by conjugation with azido-rhodamine and in-gel fluorescence analysis. Rho: rhodamine.

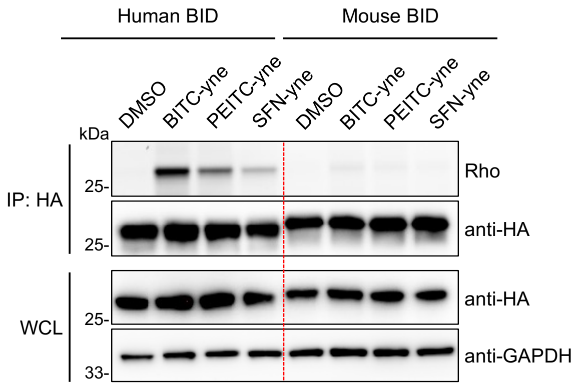

Supplementary Figure S5. In-gel fluorescence analysis of human and mouse BID labeling by ITC probes. HEK293T cells were transfected to express HA-tagged human or mouse BID, labeled with the probe (20 μM) for 0.5 h, and subjected to immunoprecipitation (IP), followed by conjugation with azido-rhodamine and in-gel fluorescence analysis. Rho: rhodamine.

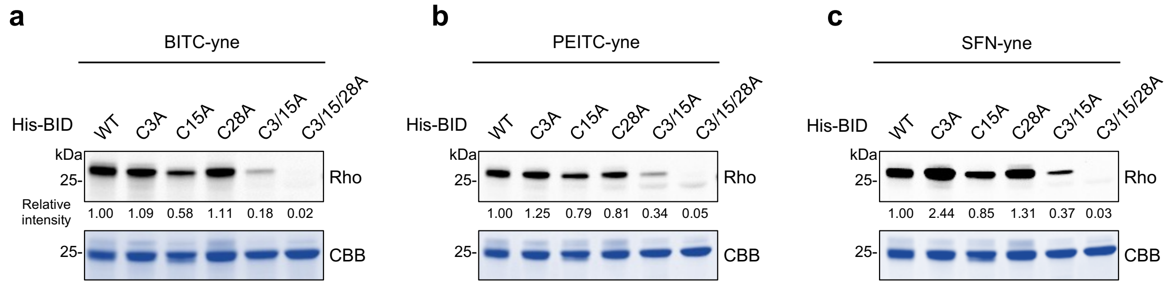

Supplementary Figure S6. In-gel fluorescence analysis of recombinant BID labeled by ITC probes *in vitro*. Purified BID protein and mutants were incubated with **a** BITC-yne (20 μM), **b** PEITC-yne (20 μM), or **c** SFN-yne (20 μM) for 2 h, conjugated with azido-rhodamine, and analyzed by in-gel fluorescence. Relative rhodamine intensities are quantified and shown. Rho: rhodamine; CBB: Coomassie Brilliant Blue staining.

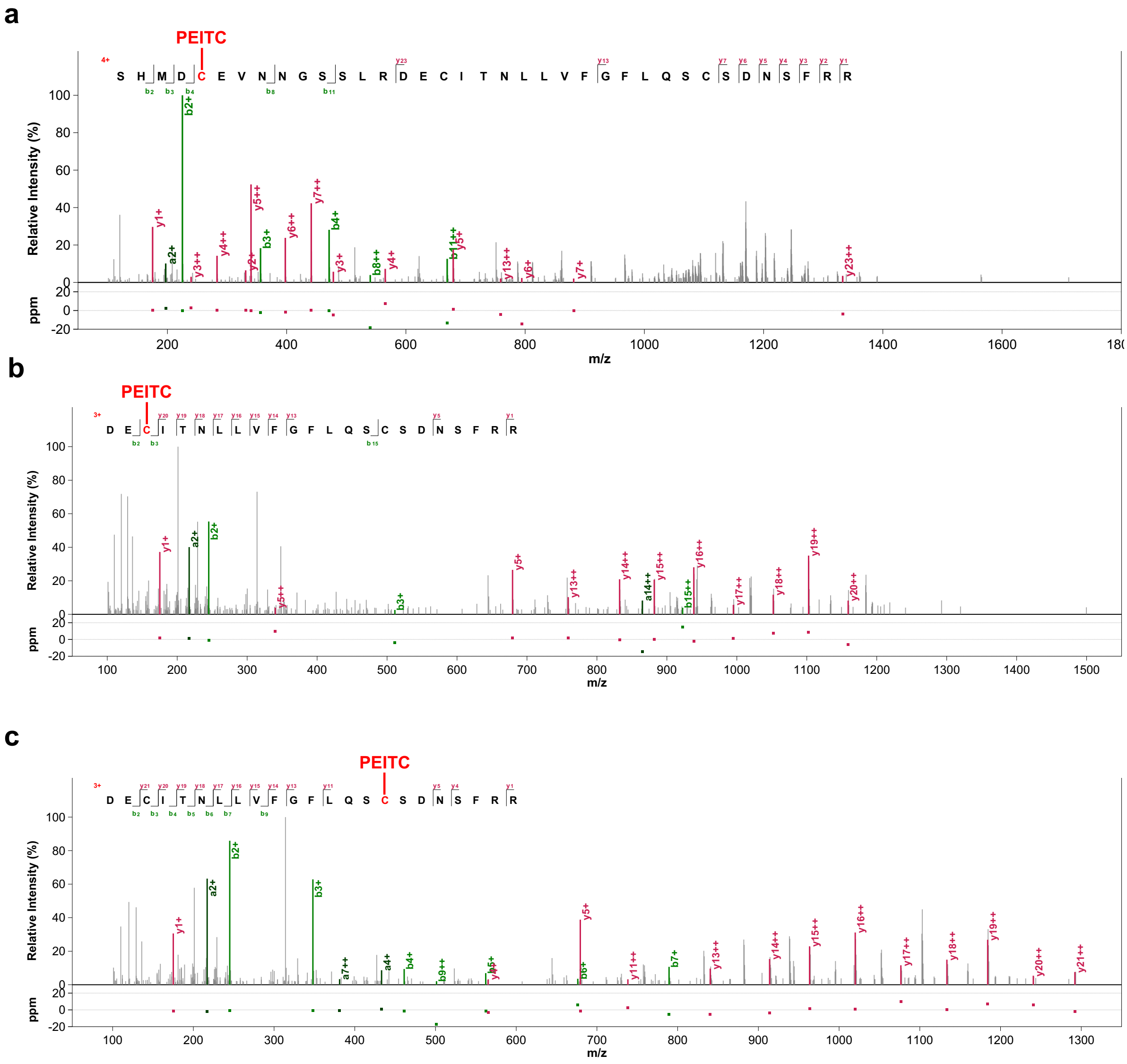

Supplementary Figure S7. LC-MS/MS analysis of BID modification sites. a Representative MS/MS spectrum showing the covalent modification of PEITC on C3 of BID. **b** Representative MS/MS spectrum showing the covalent modification of PEITC on C15 of BID. **c** Representative MS/MS spectrum showing the covalent modification of PEITC on C28 of BID. The recombinant protein (15 μg) was incubated with PEITC (20 μM) in the presence of TCEP (5 μM) at room temperature in the dark for 2 h and digested with trypsin at 37 °C for 2 h. The resulting peptides were analyzed by LC-MS/MS.

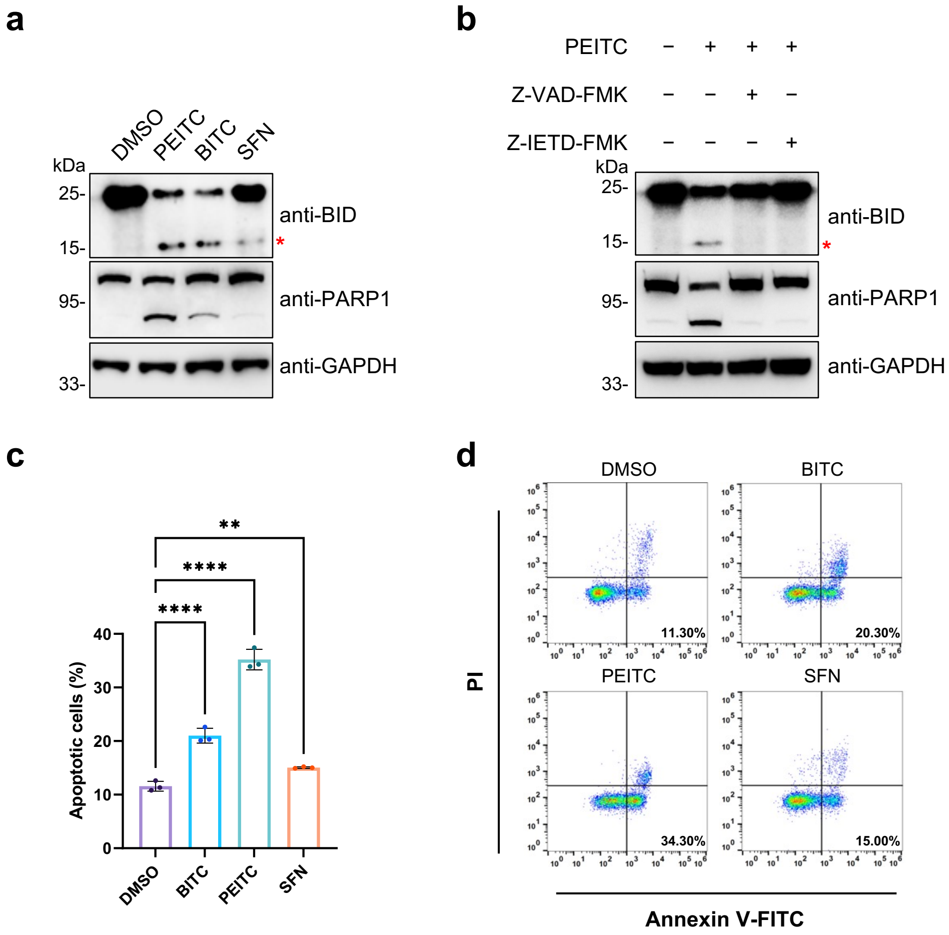

Supplementary Figure S8. ITCs induce the cleavage of BID and apoptosis in MCF-7 cells. **a** Western blotting analysis of endogenous BID cleavage induced by ITCs. MCF-7 cells were incubated with ITCs (20 μM) for 4 h. **b** MCF-7 cells were incubated with PEITC (20 μM) for 4 h in the absence or presence of Z-VAD-FMK (20 μM) or Z-IETD-FMK (20 μM). **c** Percentages of apoptotic cells measured by Annexin V staining and flow cytometry. MCF-7 cells were treated with ITCs (20 μM) for 4 h. Data are shown as mean ± sd (*n* = 3). Statistical analysis was performed with one-way ANOVA test (** $p < 0.01$, **** $p < 0.0001$). **d** Representative flow cytometry data of **c** showing that ITCs induce cell apoptosis.

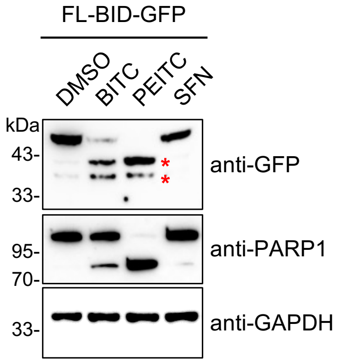

Supplementary Figure S9. ITCs induce the cleavage of BID and PARP1 in HeLa cells. HeLa cells expressing GFP-tagged BID were incubated with ITCs (20 μM) for 4 h and lysed for Western blotting analysis with antibodies against GFP and PARP1.

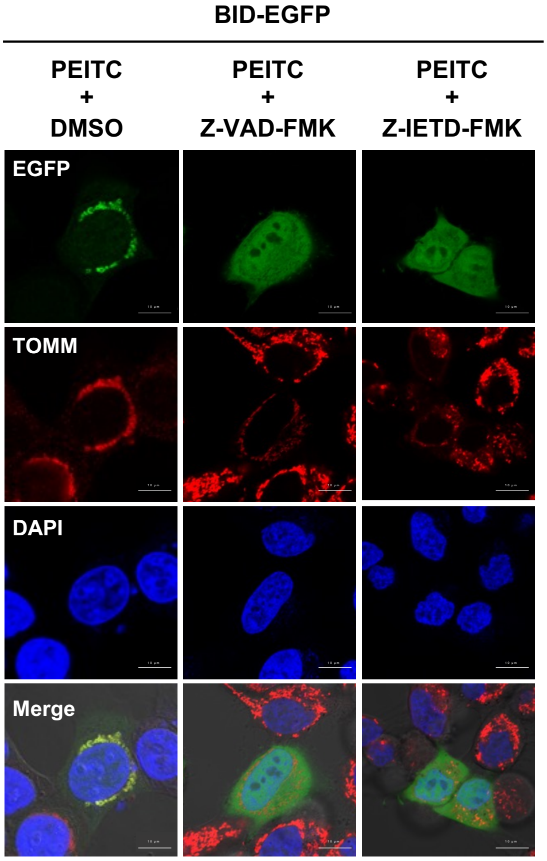

Supplementary Figure S10. Inhibition of caspase activities blocked PEITC-induced translocation of BID to mitochondria. HeLa cells expressing GFP-tagged full-length BID (BID-EGFP) and TOMM-mCherry were treated with PEITC (20 μM) in the absence or presence of Z-VAD-FMK (20 μM) or Z-IETD-FMK (20 μM) for 4 h and imaged by confocal fluorescence microscopy. Scale bars = 10 μm.

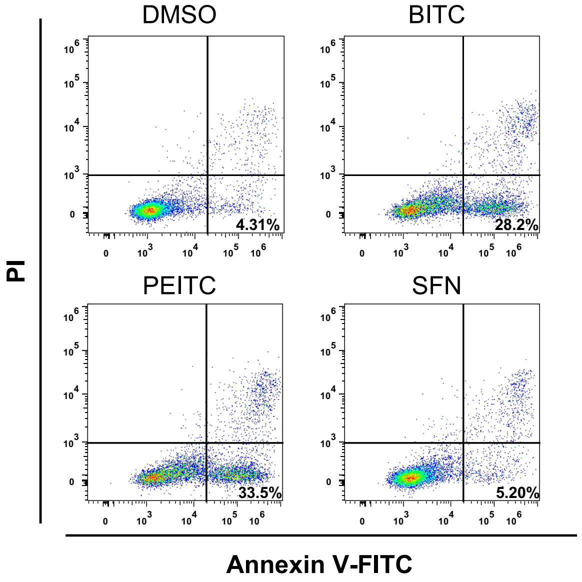

Supplementary Figure S11. Representative flow cytometry data showing that ITCs induce cell apoptosis. HeLa cells were incubated with ITCs (20 μM) for 4 h. Apoptotic cells were measured by Annexin V/PI staining and flow cytometry.

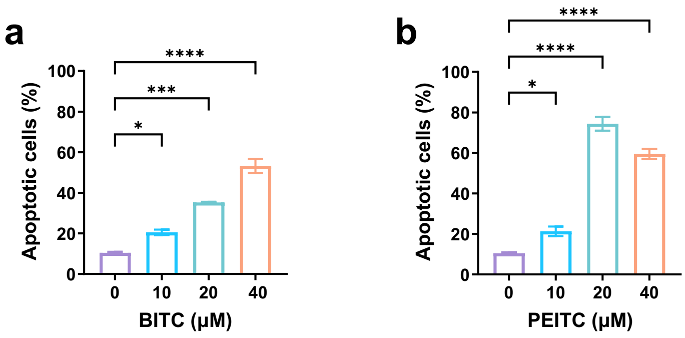

Supplementary Figure S12. ITCs induce cell apoptosis dose-dependently. HeLa cells were incubated with **a** BITC or **b** PEITC at indicated concentrations for 4 h. Apoptotic cells were measured by Annexin V/PI staining and flow cytometry. Data are shown as mean ± sd (n = 3). Statistical analyses were performed with one-way ANOVA test (* $p < 0.05$, *** $p < 0.001$, **** $p < 0.0001$).

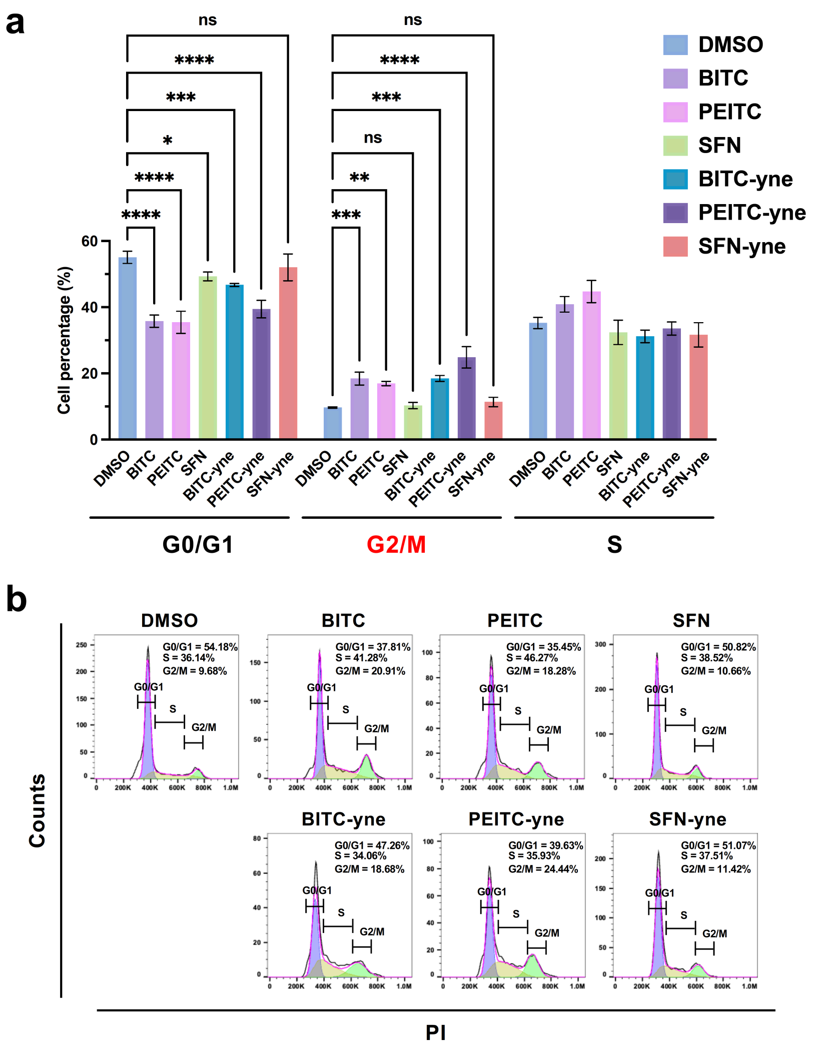

Supplementary Figure S13. Natural ITCs and ITC probes induce cell cycle arrest at G2/M in HeLa cells. a HeLa cells were treated with natural ITCs or ITC probes (2 μM) for 72 h and stained with propidium iodide (PI) for flow cytometry analysis. Cell percentages in different phases (G0/G1 and G2/M) of cell cycle after treatment with natural ITCs or ITC probes are shown. Data are mean ± sd (n = 3). Statistical analysis was performed with two-way ANOVA test (ns $p > 0.05$ non-significant, $*$ $p < 0.05$, $**$ $p < 0.01$, $***$ $p < 0.001$, $****$ $p < 0.0001$). **b** Representative flow cytometry data of **a** showing that natural ITCs and ITC probes induce cell cycle arrest at G2/M.

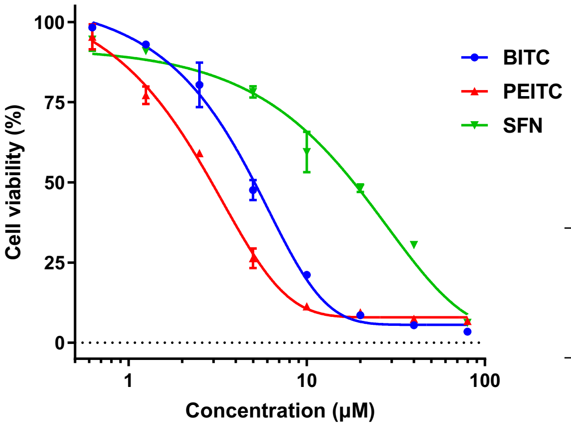

Compounds	GI_{50} (μM)
BITC	4.97
PEITC	2.93
SFN	15.17

Supplementary Figure S14. Cytotoxicity of ITCs in HeLa cells. HeLa cells were incubated with natural ITCs for 72 h and measured for viability by the MTT assay. Data are shown as mean ± sd (*n* = 3).

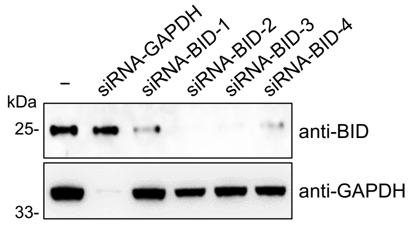

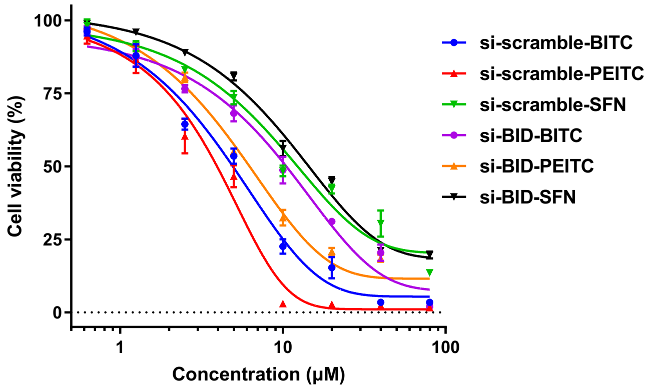

c

Compounds	GI_{50} (μM)	
	Scramble siRNA	BID siRNA
BITC	4.73	9.26
PEITC	3.58	6.50
SFN	13.10	15.20

Supplementary Figure S15. Knockout of BID affects the cytotoxicity of ITCs in HeLa cells. a Western blotting analysis of knockout of BID in HeLa cells using siRNA. Cells were transfected with the indicated siRNA for 48 h and analyzed by Western blotting. Four siRNAs (siRNA-BID-1, siRNA-BID-2, siRNA-BID-3, and siRNA-BID-4) for BID were tested. **b** and **c** Cytotoxicity of ITCs in HeLa cells with BID knockout. HeLa cells were transfected with the indicated siRNA for 48 h, treated with ITCs for 72 h, and measured for viability by the MTT assay. Data are shown as mean ± sd (*n* = 3).

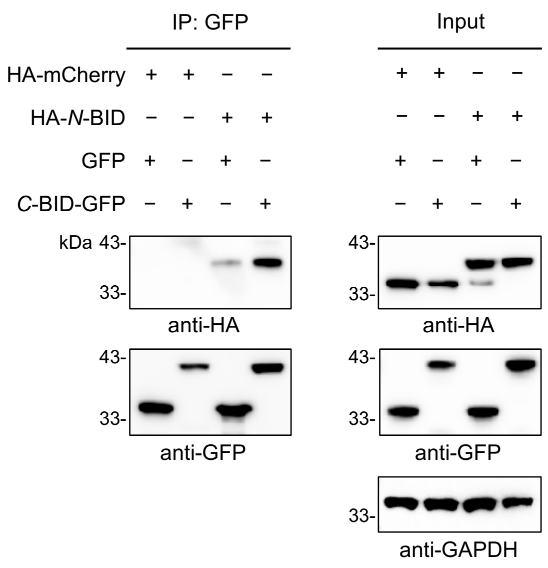

Supplementary Figure S16. Analysis of the interaction between *N*-BID and *C*-BID. HEK293T cells were transfected to express the indicated proteins, lysed, and subjected to anti-GFP immunoprecipitation. Co-immunoprecipitated HA-tagged *N*-BID was detected by Western blotting.

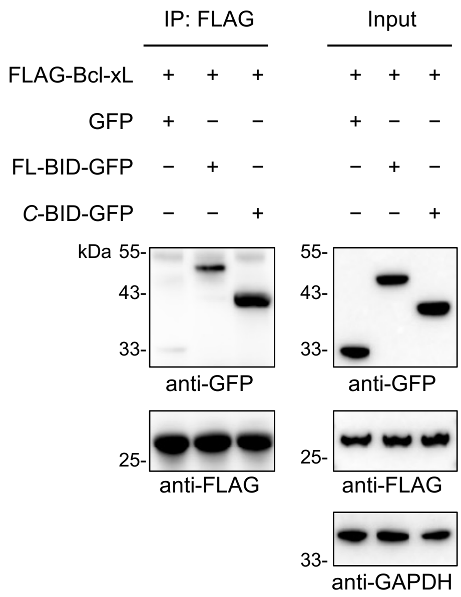

Supplementary Figure S17. Analysis of the interaction between Bcl-xL and BID. HEK293T cells were transfected to express the indicated proteins, lysed, and subjected to anti-FLAG immunoprecipitation. Co-immunoprecipitated GFP-tagged *C*-BID and FL-BID were detected by Western blotting.

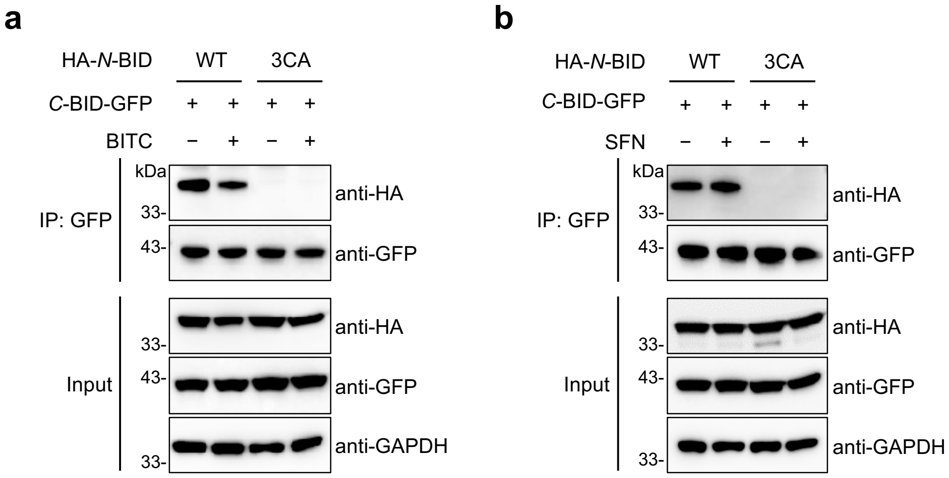

Supplementary Figure S18. The effects of BITC and SFN on the interaction between *N*-BID and *C*-BID. a Co-immunoprecipitation analysis of the effects of BITC and cysteine mutations on the interaction between *N*-BID and *C*-BID. **b** Co-immunoprecipitation analysis of the effects of SFN and cysteine mutations on the interaction between *N*-BID and *C*-BID. HEK293T cells expressing the indicated proteins were treated with BITC or SFN (20 μM) for 4 h and lysed for anti-GFP immunoprecipitation and Western blotting detection of co-immunoprecipitated HA-tagged *N*-BID.

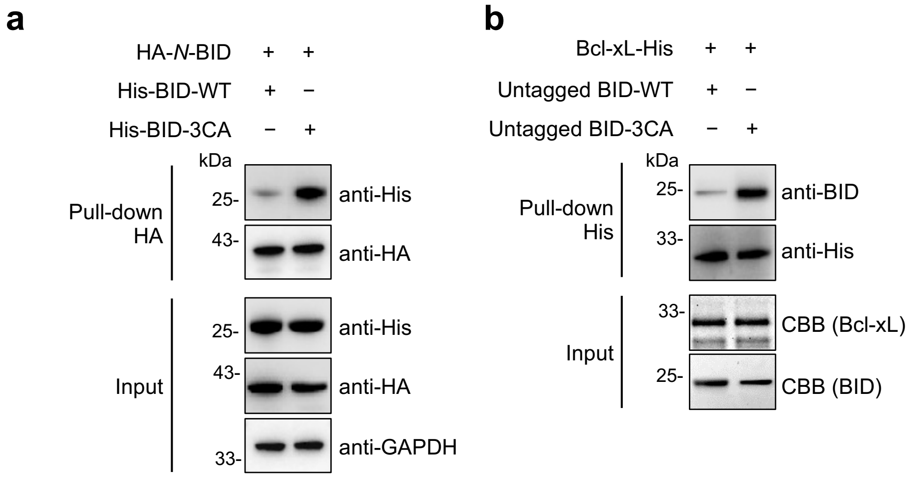

Supplementary Figure S19. The effects of cysteine mutations on BID interactions. a *In vitro* pull-down analysis of the effects of cysteine mutations on the association between *N*-BID and FL-BID. Lysates from HEK293T cells expressing HA-tagged *N*-BID were incubated with recombinant His-tagged BID for 1 h and subjected to anti-HA pull-down, followed by Western

blotting analysis. **b** *In vitro* pull-down analysis of the effects of cysteine mutations on the association between Bcl-xL and BID. Recombinant BID was incubated with recombinant His-tagged Bcl-xL for 0.5 h and subjected to His-tag pull-down, followed by Western blotting analysis with an anti-BID antibody.

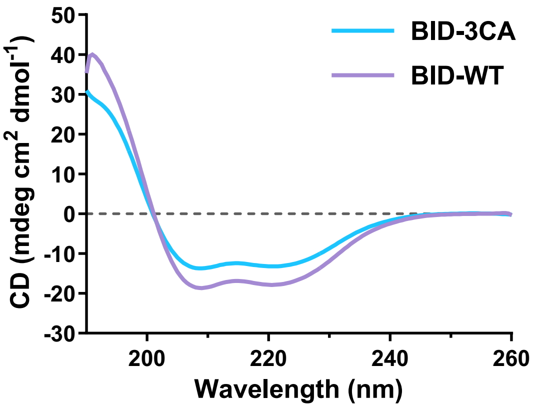

Supplementary Figure S20. Secondary structural changes of BID upon cysteine mutations. The CD spectra of recombinant BID and 3CA mutant (C3/15/28A) (5 μM) were recorded.

Chemical Synthesis

General methods and materials

Chemicals and solvents were generally purchased from commercial sources (TCI, Bidepharm, and Leyan) and used directly as received without further purification unless otherwise noted. Chemical reactions were performed in oven-dried flasks under a N_2 or Ar atmosphere when necessary. Anhydrous dichloromethane (DCM) was distilled from calcium hydride. Anhydrous tetrahydrofuran (THF) was distilled from sodium/benzophenone. TLC was conducted on silica gel 60 GF254 glass plates (Qingdao Haiyang Chemical Co., Ltd) and spots were visualized by illumination with a 254 nm UV light and/or staining with phosphomolybdic acid (PMA). Flash column chromatography was performed with silica gel (230-400 mesh, reagent grade; Qingdao Haiyang Chemical Co., Ltd). ^{1}H and ^{13}C NMR spectra were recorded in $CDCl_3$ or CD_3OD at room temperature on Bruker Avance NMR Spectrometers operating at 300 MHz, 400 MHz, or 500 MHz for ^{1}H. Chemical shifts are reported in δ ppm and coupling constants (*J* values) are reported in Hz. ^{1}H NMR chemical shifts are calibrated using tetramethylsilane (TMS, δ = 0.00 ppm) in $CDCl_3$ as the internal standard. ^{13}C NMR chemical shifts are calibrated using $CDCl_3$ (δ = 77.16 ppm) or CD_3OD (δ = 49.00 ppm) as the internal standard. High resolution ESI mass spectra were recorded with an Q Exactive Focus (ThermoFisher) mass spectrometer.

Synthesis of BITC-yne

Scheme S1. Synthesis of BITC-yne

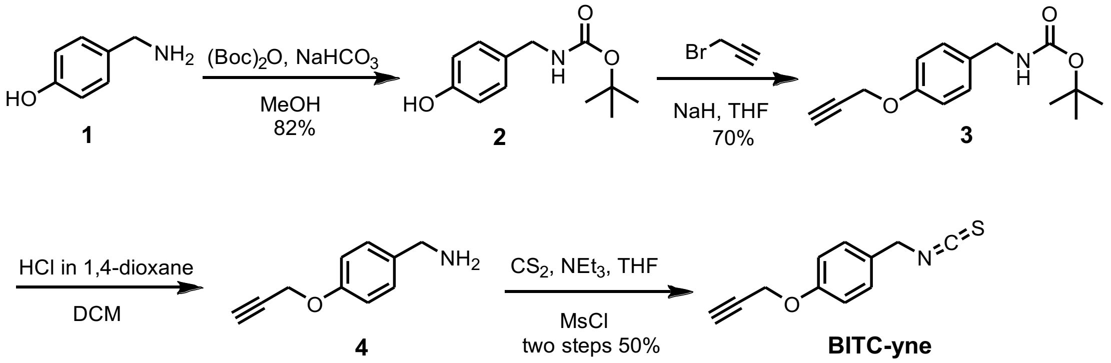

Synthesis of tert-butyl (4-hydroxybenzyl)carbamate (compound 2). Compound **2** was synthesized according to procedures in a previous report.[1] To a solution of di-tert-butyl

dicarbonate (Boc_2O, 2.05 mL, 8.93 mmol) in MeOH (50 mL) was added 4-(aminomethyl) phenol (1 g, 8.12 mmol) and $NaHCO_3$ (2.73 g, 32.48 mmol). The reaction mixture was stirred at room temperature overnight. After that, the mixture was filtered to remove extra $NaHCO_3$ and concentrated under reduced pressure. The residue was purified by silica gel column chromatography (ethyl acetate/hexane = 1:10) to afford compound **2** (1.49 g, 82% yield). The NMR spectra are consistent with those reported in the literature.[1] ^{1}H NMR (500 MHz, $CDCl_3$) δ 7.08 (d, *J* = 8.0 Hz, 2H), 6.80 (d, *J* = 8.0 Hz, 2H), 5.05 (s, 1H), 4.21 (d, *J* = 5.6 Hz, 2H), 1.48 (s, 9H).

Synthesis of tert-butyl (4-(prop-2-yn-1-yloxy)benzyl)carbamate (compound 3). To a stirring solution of compound **2** (1.49 g, 6.66 mmol) in THF (50 mL) at 0 °C was added NaH (400 mg, 9.99 mmol). After stirring for 15 min, propargyl bromide (1.15 mL, 13.33 mmol) was added into the reaction mixture dropwise at 0 °C. The resulting mixture was warmed to ambient temperature and stirred overnight. The reaction was quenched with NH_4Cl and extracted with ethyl acetate (3 × 20 mL). The combined organic layer was washed with brine (20 mL), dried over anhydrous Na_2SO_4, and concentrated under reduced pressure. The residue was purified by silica gel column chromatography (ethyl acetate/hexane = 1:15) to afford compound **3** (1.22 g, 70% yield). ^{1}H NMR (300 MHz, $CDCl_3$) δ 7.23 (d, *J* = 8.5 Hz, 2H), 6.94 (d, *J* = 8.5 Hz, 2H), 4.68 (d, *J* = 2.4 Hz, 2H), 4.26 (d, *J* = 5.8 Hz, 2H), 2.52 (t, *J* = 2.4 Hz, 1H), 1.46 (s, 9H). ^{13}C NMR (75 MHz, $CDCl_3$) δ 156.80, 155.83, 132.00, 128.82, 114.99, 79.46, 78.51, 75.55, 55.83, 44.11, 28.42. HRMS (ESI) m/z calculated for $C_{15}H_{19}NO_3Na^+$ $[M+Na]^+$: 284.1257, found: 284.1258.

Synthesis of 1-(isothiocyanatomethyl)-4-(prop-2-yn-1-yloxy)benzene (BITC-yne). To a solution of compound **3** (1.22 g, 4.67 mmol) in CH_2Cl_2 (50 mL) was added 4 M HCl in 1,4-dioxane (7 mL) dropwise. The reaction mixture was stirred at room temperature for 5 h. After that, the mixture was concentrated under reduced pressure to afford compound **4** as the crude product, which was used directly without further purification. Compound **4** was dissolved in THF (30 mL). To this solution was added triethylamine (2.59 mL, 18.68 mmol) and carbon disulfide (CS_2, 0.31 mL, 5.14 mmol) dropwise at 0 °C with vigorous stirring. The reaction

mixture was warmed to ambient temperature and stirred for 90 min. After that, methanesulfonyl chloride (MsCl, 0.40 mL, 5.14 mmol) was added at 0 °C. The reaction mixture was stirred at room temperature for another 30 min, diluted with CH_2Cl_2, and washed with 1N HCl and brine. The organic layer was evaporated under reduced pressure and the resulting residue was purified by silica gel column chromatography (ethyl acetate/hexane = 1:10) to afford **BITC-yne** (474.6 mg, 50% yield for two steps). ^{1}H NMR (400 MHz, $CDCl_3$) δ 7.26 (d, *J* = 8.7 Hz, 2H), 7.00 (d, *J* = 8.7 Hz, 2H), 4.71 (d, *J* = 2.4 Hz, 2H), 4.66 (s, 2H), 2.54 (t, *J* = 2.4 Hz, 1H). ^{13}C NMR (101 MHz, $CDCl_3$) δ 157.52, 132.30, 128.35, 127.28, 115.34, 78.24, 75.76, 55.87, 48.21. HRMS (ESI) m/z calculated for $C_{11}H_8NOS^-$ $[M–H]^-$: 202.0332, found: 202.0327.

Synthesis of PEITC-yne

Scheme S2. Synthesis of PEITC-yne

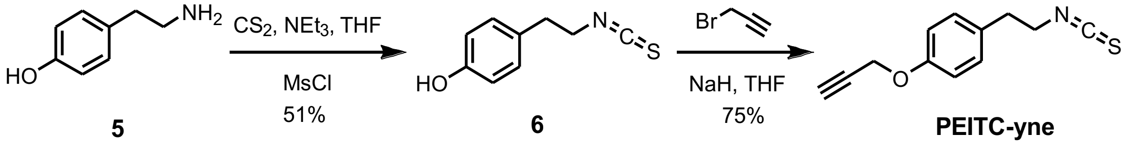

Synthesis of 4-(2-isothiocyanatoethyl)phenol (compound 6). To a vigorously stirring solution of 4-(2-aminoethyl)phenol (**5**) (500 mg, 3.64 mmol) in THF (20 mL) was added triethylamine (1.61 mL, 11.65 mmol) and carbon disulfide (CS_2, 0.24 mL, 4.01 mmol) dropwise at 0 °C. The reaction mixture was warmed to ambient temperature and stirred for 90 min. After that, methanesulfonyl chloride (MsCl, 0.35 mL, 4.01 mmol) was added at 0 °C. The reaction mixture was stirred at room temperature for another 30 min, diluted with CH_2Cl_2, and washed with 1N HCl and brine. The organic layer was evaporated under reduced pressure and the resulting residue was purified by silica gel column chromatography (ethyl acetate/hexane = 1:10) to afford compound **6** (333.4 mg, 51% yield). ^{1}H NMR (300 MHz, $CDCl_3$) δ 7.08 (d, *J* = 8.5 Hz, 2H), 6.82 (d, *J* = 8.5 Hz, 2H), 5.47 (s, 1H), 3.67 (t, *J* = 6.9 Hz, 2H), 2.91 (t, *J* = 6.9 Hz, 2H). ^{13}C NMR (75 MHz, $CDCl_3$) δ 172.36, 154.67, 130.07, 129.17, 115.68, 46.64, 35.65. HRMS (ESI) m/z calculated for $C_9H_8NOS^-$ $[M–H]^-$: 178.0332, found: 178.0325.

Synthesis of 1-(2-isothiocyanatoethyl)-4-(prop-2-yn-1-yloxy)benzene (PEITC-yne). To a

solution of compound **6** (100 mg, 0.56 mmol) in THF (10 mL) at 0 °C was added NaH (40 mg, 1.68 mmol). After stirring for 15 min, propargyl bromide (0.14 mL, 1.68 mmol) was added into the reaction mixture dropwise at 0 °C. The resulting mixture was warmed to ambient temperature and stirred overnight. The reaction was quenched with NH_4Cl and extracted with ethyl acetate (3 × 20 mL). The combined organic layer was washed with brine (20 mL), dried over anhydrous Na_2SO_4, and concentrated under reduced pressure. The residue was purified by silica gel column chromatography (ethyl acetate/hexane = 1:10) to afford **PEITC-yne** (91 mg, 75% yield). ^{1}H NMR (400 MHz, $CDCl_3$) δ 7.16 (d, *J* = 8.6 Hz, 2H), 6.96 (d, *J* = 8.6 Hz, 2H), 4.70 (d, *J* = 2.4 Hz, 2H), 3.70 (t, *J* = 7.0 Hz, 2H), 2.95 (t, *J* = 6.9 Hz, 2H), 2.53 (t, *J* = 2.4 Hz, 1H). ^{13}C NMR (101 MHz, $CDCl_3$) δ 156.72, 138.45, 130.00, 129.83, 115.22, 78.49, 75.54, 55.85, 46.54, 35.70. HRMS (ESI) m/z calculated for $C_{12}H_{10}NOS^-$ $[M–H]^-$: 216.0489, found: 216.0482.

Synthesis of SFN-yne

Scheme S3. Synthesis of SFN-yne

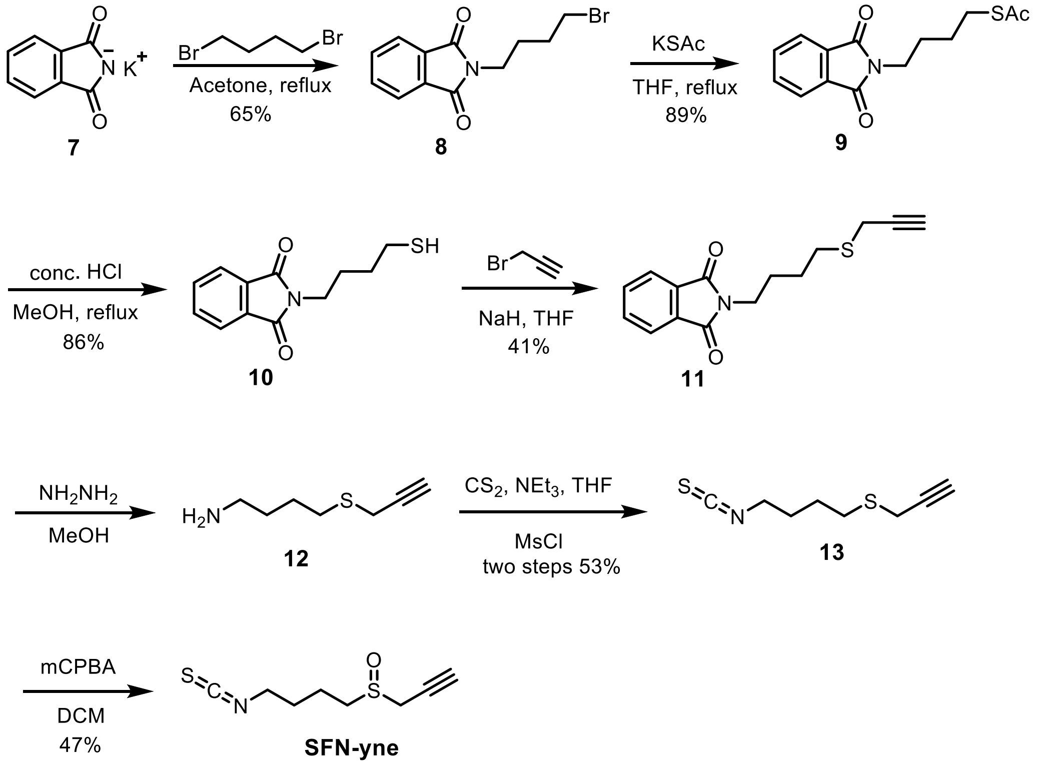

Synthesis of 2-(4-bromobutyl)isoindoline-1,3-dione (compound 8). Compound **8** was

synthesized according to procedures in a previous report.[2] To a stirring solution of 1,4-dibromobutane (0.97 mL, 8.10 mmol) in acetone (40 mL) was added potassium phthalimide (**7**) (500 mg, 2.70 mmol) slowly over 15 min. The reaction mixture was heated to reflux overnight, cooled to room temperature, and filtered. The filtrate was concentrated under reduced pressure and the residue was purified by silica gel column chromatography (ethyl acetate/hexane = 1:50) to afford compound **8** (490 mg, 65% yield). The NMR spectra are consistent with those reported in the literature.[2] ^{1}H NMR (500 MHz, $CDCl_3$) δ 7.87 (dd, *J* = 5.4, 3.0 Hz, 2H), 7.74 (dd, *J* = 5.5, 3.0 Hz, 2H), 3.75 (t, *J* = 6.7 Hz, 2H), 3.47 (t, *J* = 6.4 Hz, 2H), 1.99–1.81 (m, 4H).

Synthesis of *S*-(4-(1,3-dioxoisoindolin-2-yl)butyl)ethanethioate (compound 9). Compound **9** was synthesized according to procedures in a previous report.[2] To a stirring solution of compound **8** (490 mg, 1.74 mmol) in THF (20 mL) was added potassium thioacetate (597 mg, 5.23 mmol). The mixture was heated to reflux for 4 h and then concentrated under reduced pressure. The residue was diluted with ethyl acetate, washed with water and brine, dried over anhydrous $MgSO_4$, and concentrated under reduced pressure. The crude product was purified by silica gel column chromatography (ethyl acetate/hexane = 1:20) to give compound **9** (430 mg, 89% yield). The NMR spectra are consistent with those reported in the literature.[2] ^{1}H NMR (300 MHz, $CDCl_3$) δ 7.83 (dd, *J* = 5.5, 3.1 Hz, 2H), 7.70 (dd, *J* = 5.4, 3.1 Hz, 2H), 3.68 (t, *J* = 7.0 Hz, 2H), 2.89 (t, *J* = 7.0 Hz, 2H), 2.30 (s, 3H), 1.78–1.71 (m, 2H), 1.69–1.54 (m, 2H).

Synthesis of 2-(4-mercaptobutyl)isoindoline-1,3-dione (compound 10). Compound **10** was synthesized according to procedures in a previous report.[2] A solution of compound **9** (430 mg, 1.55 mmol) in anhydrous methanol (20 mL) was degassed and refilled with N_2. Concentrated HCl (0.62 mL) was added into the mixture slowly and the reaction was heated to reflux for 5 h. After that, the reaction was quenched with water and extracted with ethyl acetate. The organic layer was dried over anhydrous $MgSO_4$ and concentrated under reduced pressure. The residue was purified by silica gel column chromatography (ethyl acetate/hexane = 1:20) to give compound **10** (316 mg, 86% yield). The NMR spectra are consistent with those

reported in the literature.[2] ^1H NMR (300 MHz, $CDCl_3$) δ 7.85 (dd, *J* = 5.4, 3.1 Hz, 2H), 7.72 (dd, *J* = 5.5, 3.0 Hz, 2H), 3.71 (t, *J* = 7.0 Hz, 2H), 2.58 (dt, *J* = 7.9, 6.9 Hz, 2H), 1.89–1.76 (m, 2H), 1.72–1.63 (m, 2H).

Synthesis of 2-(4-(prop-2-yn-1-ylthio)butyl)isoindoline-1,3-dione (compound 11). To a stirring solution of compound **10** (316 mg, 1.34 mmol) in THF (10 mL) was added NaH (64 mg, 2.68 mmol) at 0 °C. After 15 min, propargyl bromide (0.14 mL, 1.68 mmol) was added into the reaction mixture dropwise at 0 °C. The resulting mixture was warmed to ambient temperature and stirred for 2 h. The reaction was quenched with NH_4Cl and extracted with ethyl acetate (3 × 20 mL). The combined organic layer was washed with brine (20 mL), dried over anhydrous Na_2SO_4, and concentrated under reduced pressure. The residue was purified by silica gel column chromatography (ethyl acetate/hexane = 1:18) to afford compound **11** (150 mg, 41% yield). ^1H NMR (400 MHz, $CDCl_3$) δ 7.84 (dd, *J* = 5.4, 3.0 Hz, 2H), 7.72 (dd, *J* = 5.5, 3.0 Hz, 2H), 3.72 (t, *J* = 7.1 Hz, 2H), 3.24 (d, *J* = 2.6 Hz, 2H), 2.73 (t, *J* = 7.2 Hz, 2H), 2.21 (t, *J* = 2.6 Hz, 1H), 1.87–1.76 (m, 2H), 1.74–1.64 (m, 2H). ^{13}C NMR (101 MHz, $CDCl_3$) δ 168.37, 133.91, 132.08, 123.20, 79.98, 70.97, 37.44, 31.01, 27.70, 26.20, 19.14. HRMS (ESI) m/z calculated for $C_{15}H_{17}NO_2S^+$ $[M+H]^+$: 274.0896, found: 274.0895.

Synthesis of (4-isothiocyanatobutyl)(prop-2-yn-1-yl)sulfane (compound 13). Compound **11** (150 mg, 0.55 mmol) in MeOH (10 mL) was mixed with 70% hydrazine hydrate (0.66 mL, 1.37 mmol, 70% in water) and the mixture was stirred at 70 °C for 4 h. After that, the solvent was removed under reduced pressure and the residue was diluted with ethyl acetate and 1 N NaOH. The organic layer was washed with water and brine, dried over anhydrous $MgSO_4$, and concentrated under reduced pressure. The resulting crude product of compound **12** was used for the next step without purification and directly dissolved in THF (10 mL). To the stirring solution of compound **12** (66 mg, 0.46 mmol) in THF was added triethylamine (0.204 mL, 1.47 mmol) and carbon disulfide (CS_2, 0.03 mL, 0.51 mmol) dropwise at 0 °C. The reaction mixture was warmed to ambient temperature and stirred for 90 min. After that, methanesulfonyl chloride (MsCl, 0.04 mL, 0.51 mmol) was added at 0 °C. The reaction mixture was stirred at room temperature for another 30 min, diluted with CH_2Cl_2, and washed with 1N HCl and brine.

The organic layer was evaporated under reduced pressure and the resulting residue was purified by silica gel column chromatography (ethyl acetate/hexane = 1:15) to afford compound **13** (50 mg, 53% yield for two steps). ^{1}H NMR (500 MHz, $CDCl_3$) δ 3.59 (t, *J* = 6.3 Hz, 2H), 3.29 (d, *J* = 2.7 Hz, 2H), 2.76 (t, *J* = 6.9 Hz, 2H), 2.28 (t, *J* = 2.6 Hz, 1H), 1.87–1.82 (m, 2H), 1.80–1.74 (m, 2H). ^{13}C NMR (126 MHz, $CDCl_3$) δ 130.91, 79.73, 71.09, 44.63, 30.70, 28.88, 25.81, 19.18. HRMS (ESI) m/z calculated for $C_8H_{12}NS_2^+$ $[M+H]^+$: 186.0406, found 186.0407.

Synthesis of 1-isothiocyanato-4-(prop-2-yn-1-ylsulfinyl)butane (SFN-yne). A solution of compound **13** (50 mg, 0.29 mmol) in CH_2Cl_2 (5 mL) was chilled to –20 °C. To this solution was added a solution of mCPBA (50 mg, 0.29 mmol) in CH_2Cl_2 (10 mL) dropwise over 5 min. The mixture was stirred at 0 °C for 1 h and the resulting light-yellow suspension was treated with saturated $NaHCO_3$ (15 mL). The organic layer was separated and the aqueous layer was extracted with CH_2Cl_2. The combined organic layers were washed with saturated $NaHCO_3$ and brine, dried with anhydrous $MgSO_4$, and concentrated under reduced pressure. The crude product was purified by silica gel column chromatography (ethyl acetate/dichloromethane = 1:10) to give **SFN-yne** (25 mg, 47%). ^{1}H NMR (400 MHz, $CDCl_3$) δ 3.62 (d, *J* = 6.9 Hz, 2H), 3.60 (d, *J* = 2.7 Hz, 2H), 2.93 (dd, *J* = 7.4, 6.6 Hz, 2H), 2.47 (t, *J* = 2.7 Hz, 1H), 2.02–1.94 (m, 2H), 1.94–1.85 (m, 2H). ^{13}C NMR (101 MHz, $CDCl_3$) δ 131.09, 76.67, 72.25, 50.39, 44.61, 42.09, 29.03, 19.83. HRMS (ESI) m/z calculated for $C_8H_{11}NOS_2Na^+$ $[M+Na]^+$: 224.0174, found: 224.0195.

References

(1) Kim, M.; Hwang, I.; Pagire, H. S.; Pagire, S. H.; Choi, W.; Choi, W. G.; Yoon, J.; Lee, W. M.; Song, J. S.; Yoo, E. K.; et al. Design, Synthesis, and Biological Evaluation of New Peripheral 5HT2A Antagonists for Nonalcoholic Fatty Liver Disease. *Journal of Medicinal Chemistry* **2020**, *63* (8), 4171-4182.
(2) Hu, K.; Qi, Y.-j.; Zhao, J.; Jiang, H.-f.; Chen, X.; Ren, J. Synthesis and biological evaluation of sulforaphane derivatives as potential antitumor agents. *European Journal of Medicinal Chemistry* **2013**, *64*, 529-539.

NMR Spectra

^{1}H NMR of **BITC-yne**

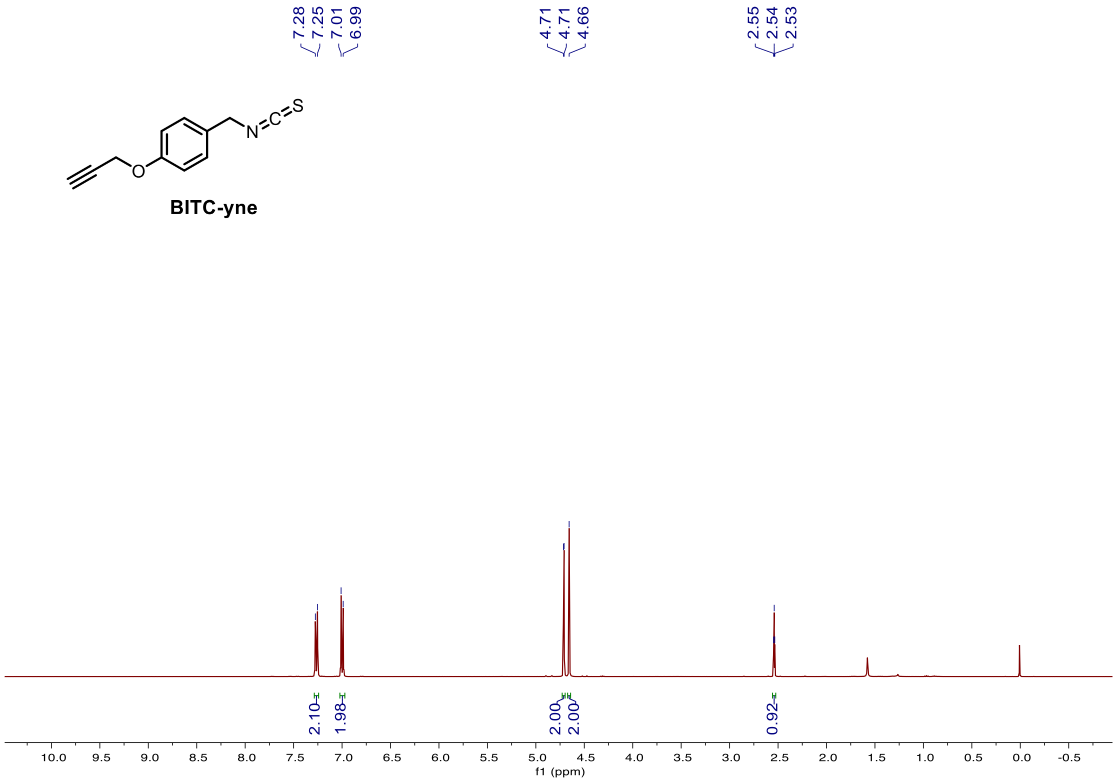

^{13}C NMR of **BITC-yne**

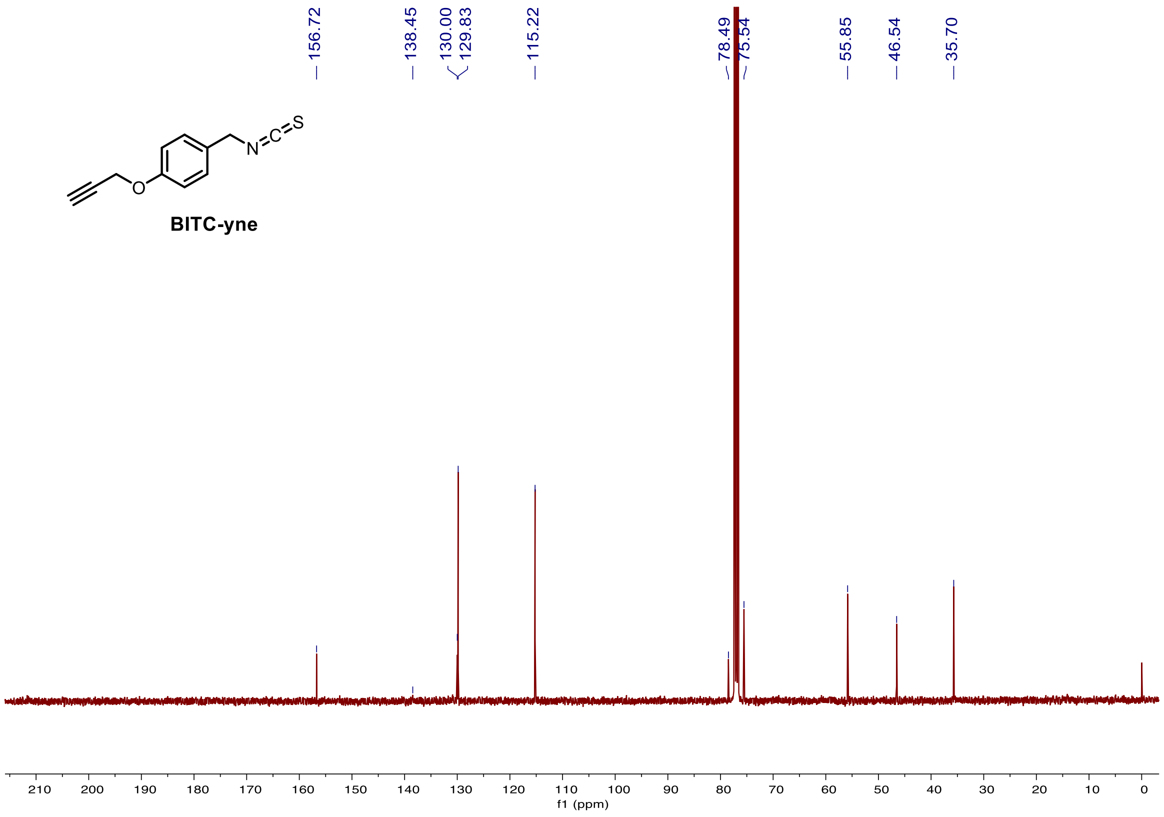

^{1}H NMR of **PEITC-yne**

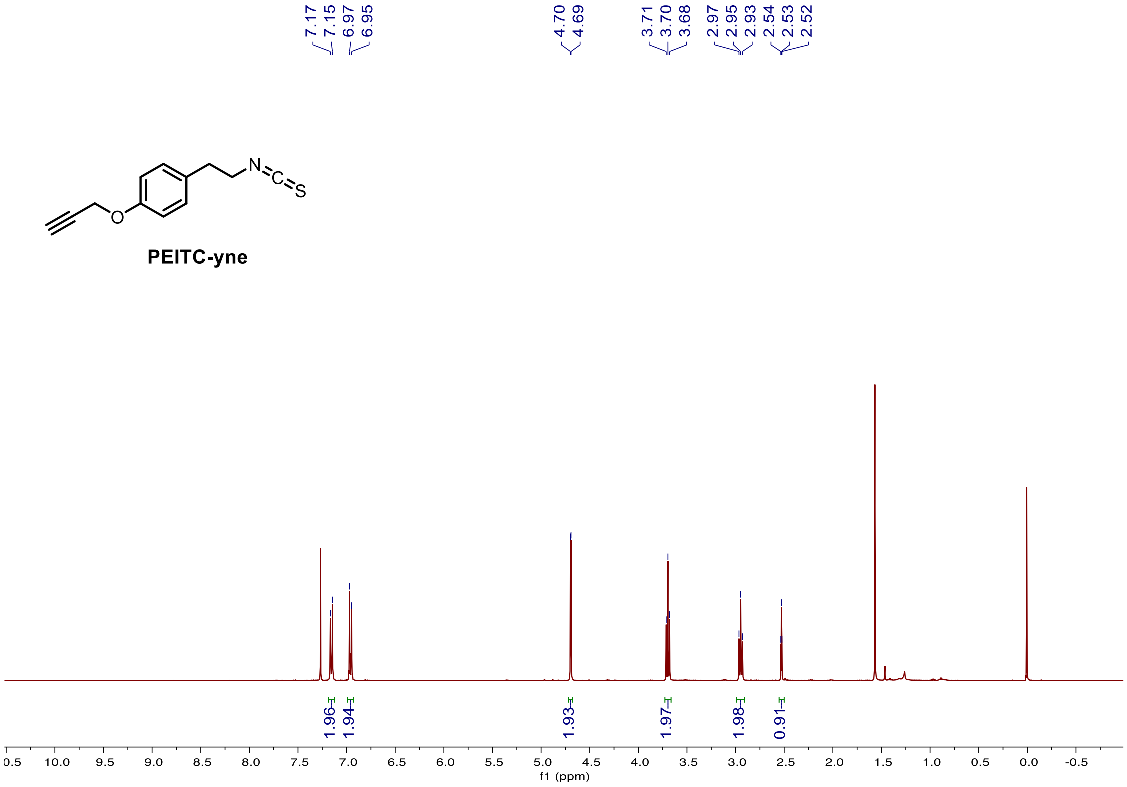

^{13}C NMR of **PEITC-yne**

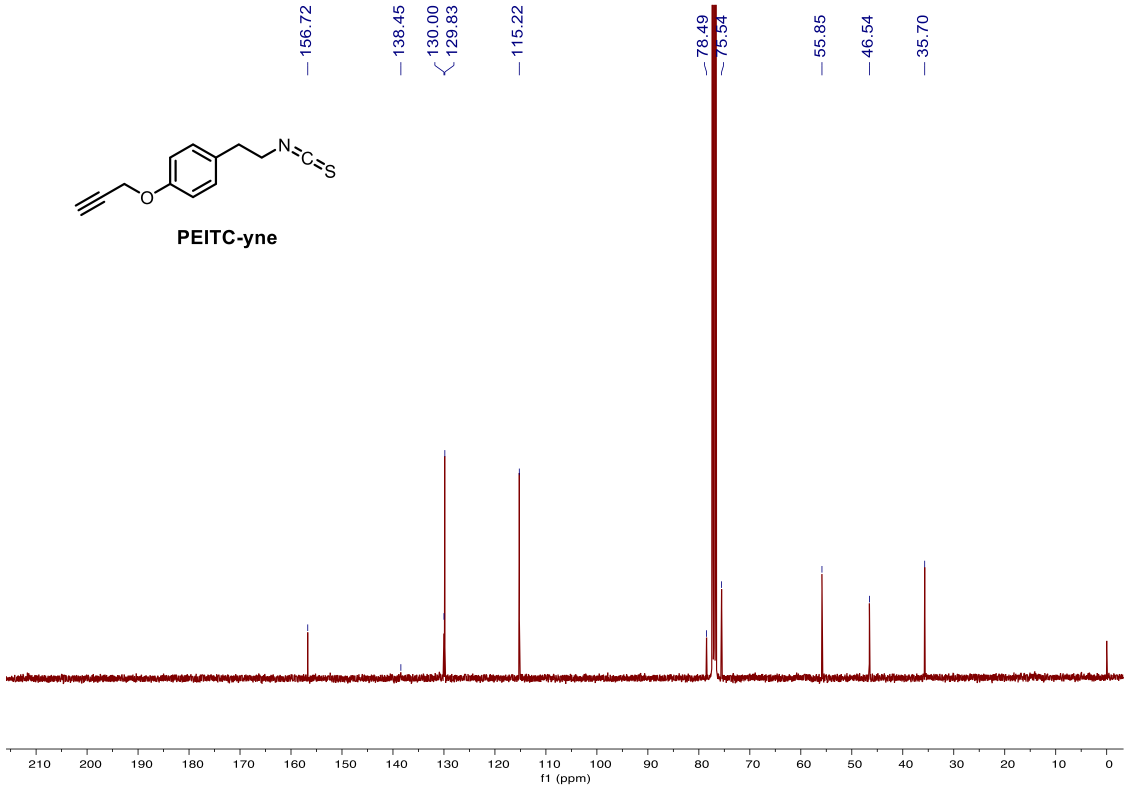

^{1}H NMR of **SFN-yne**

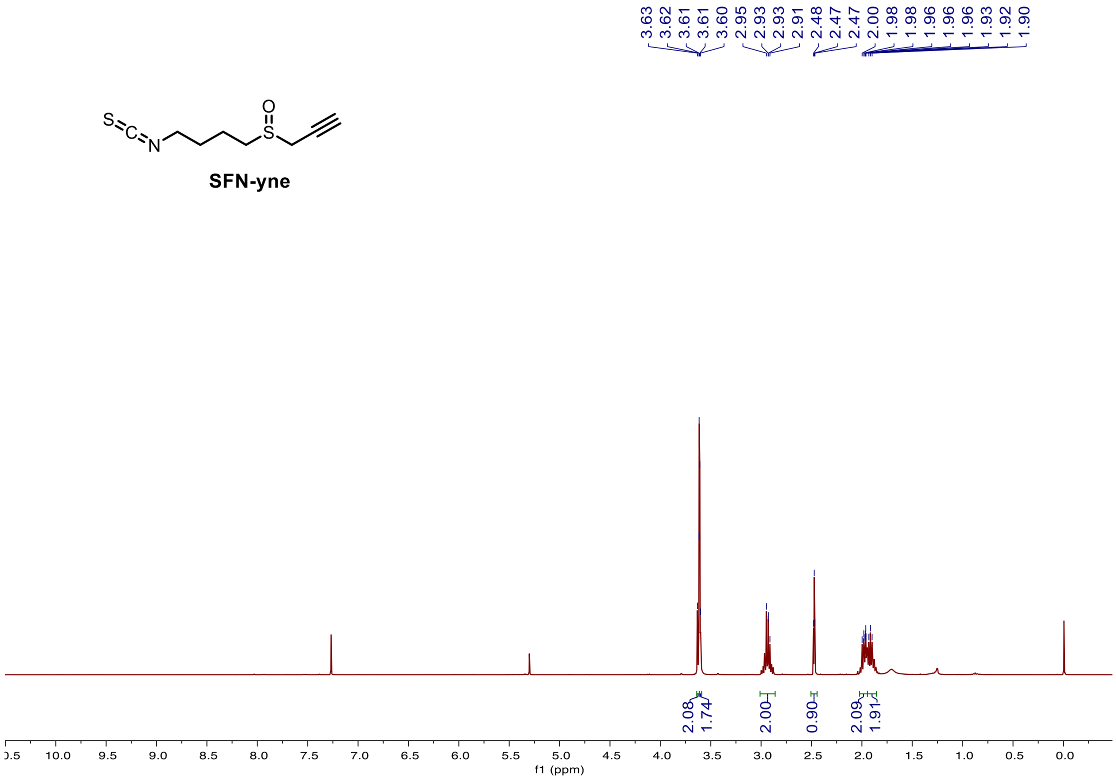

^{13}C NMR of **SFN-yne**

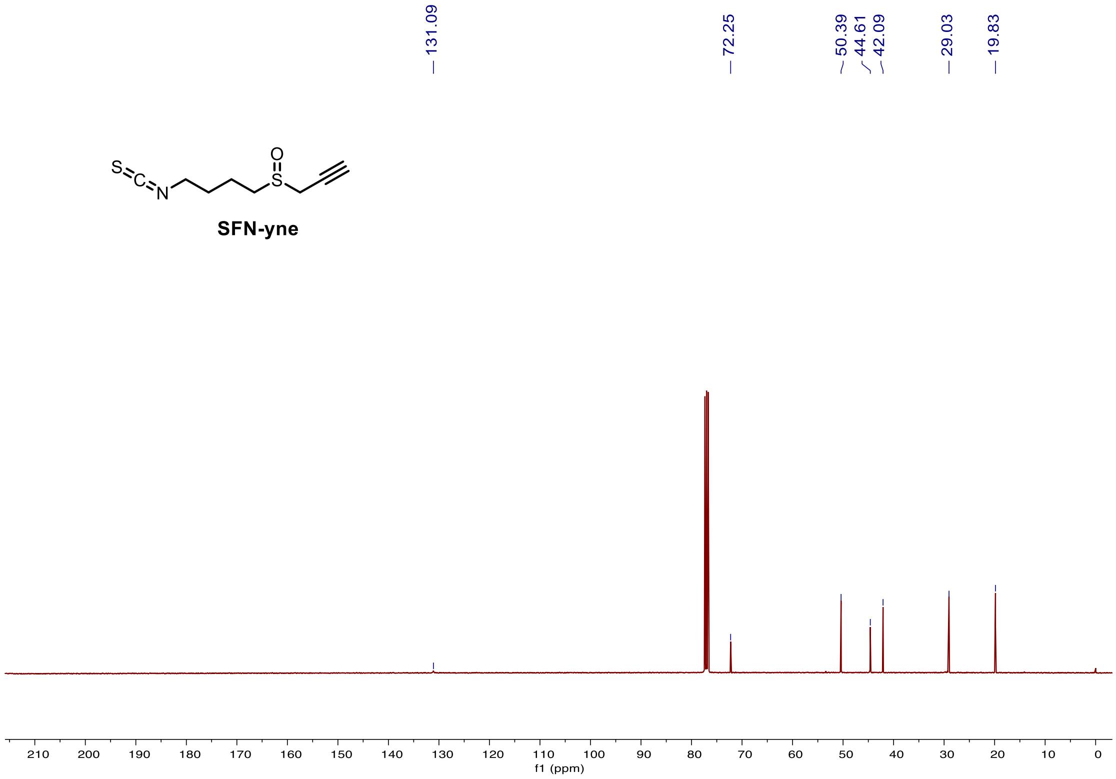

Supplement: Supplementary file 1 — Supplementary Information [file 41420_2024_2225_MOESM1_ESM.pdf]
