# Supplementary material for: Quantitative chemical proteomics reveals that phenethyl isothiocyanate covalently targets BID to promote apoptosis
Source: Cell Death Discov. 2024 Oct 29;10:456. doi: 10.1038/s41420-024-02225-7 (PMC11522290; doi:10.1038/s41420-024-02225-7)

Supplementary Information

Quantitative chemical proteomics reveal that phenethyl isothiocyanate covalently targets BID to promote apoptosis

Xiaoshu Dong[1#], Xinqian Yu[1#], Minghao Lu[1], Yaxin Xu[1], Liyan Zhou[1], and Tao Peng[1*]

[1] State Key Laboratory of Chemical Oncogenomics, School of Chemical Biology and Biotechnology, Peking University Shenzhen Graduate School, Shenzhen 518055, China

[#] These authors contributed to this work equally.

* Correspondence: Tao Peng, tpeng@pku.edu.cn

Table of Contents

Full Western Blots 2

Full Western Blots

Figure 1d

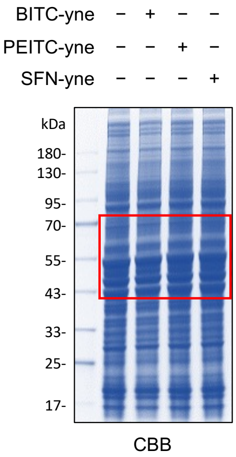

Figure 1e

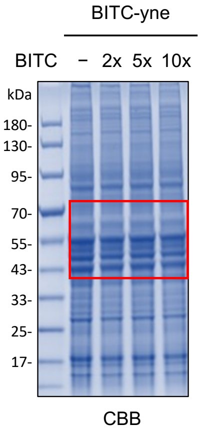

Figure 1f

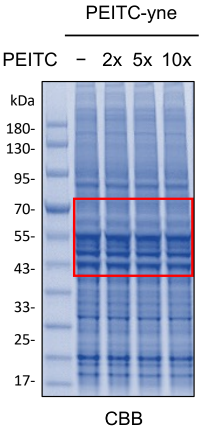

Figure 1g

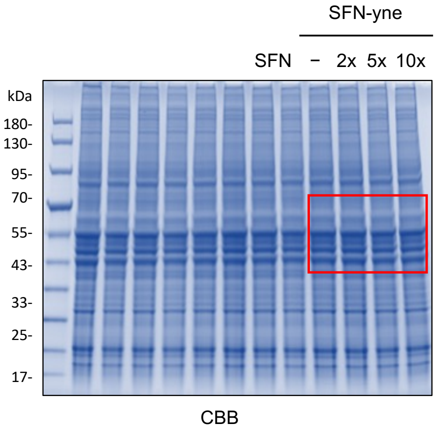

Figure 2h

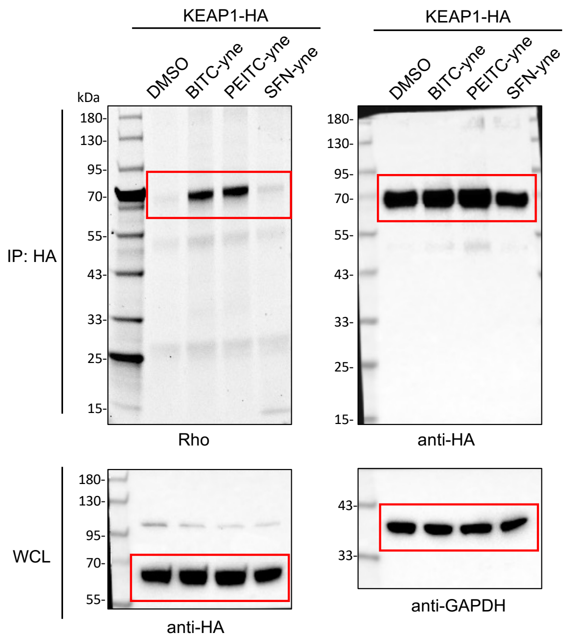

Figure 2i

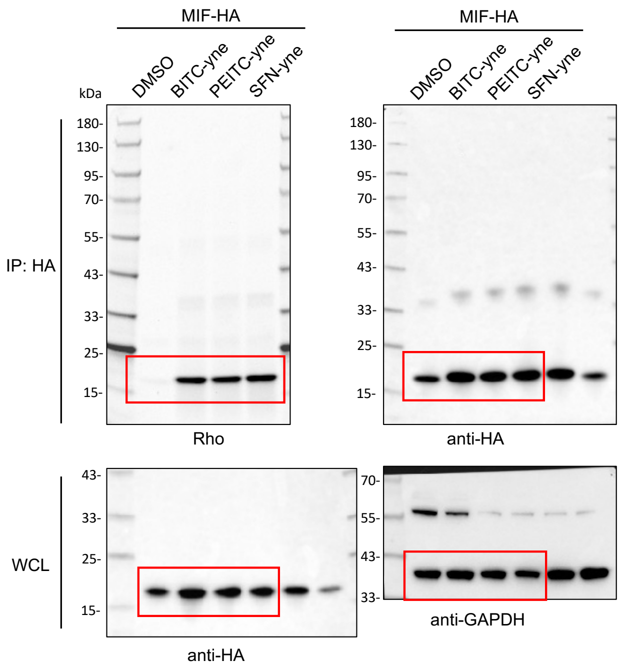

Figure 2j

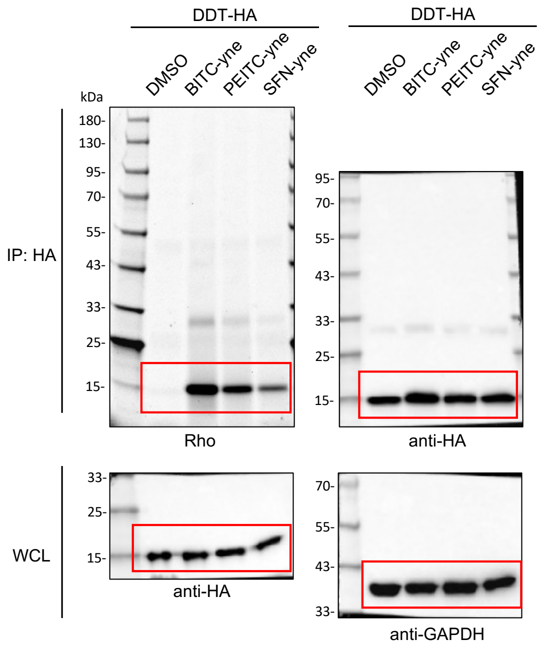

Figure 3a

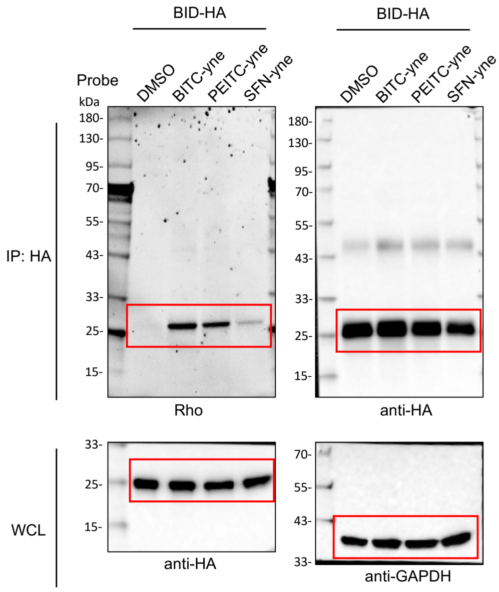

Figure 3b

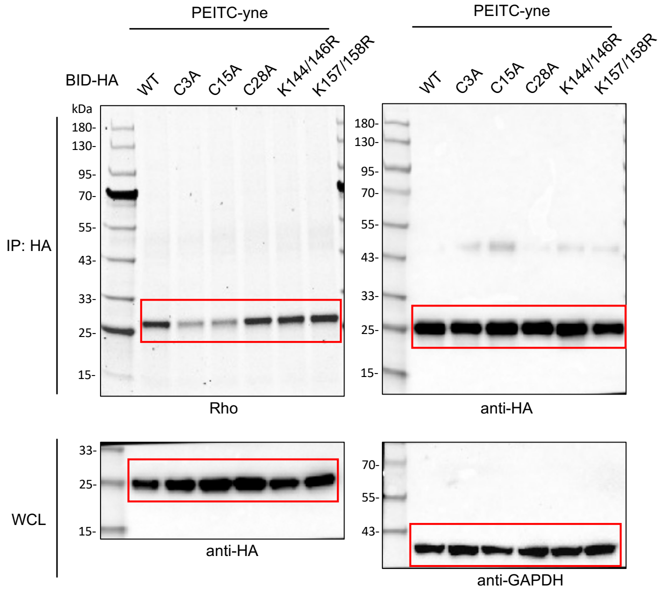

Figure 3c

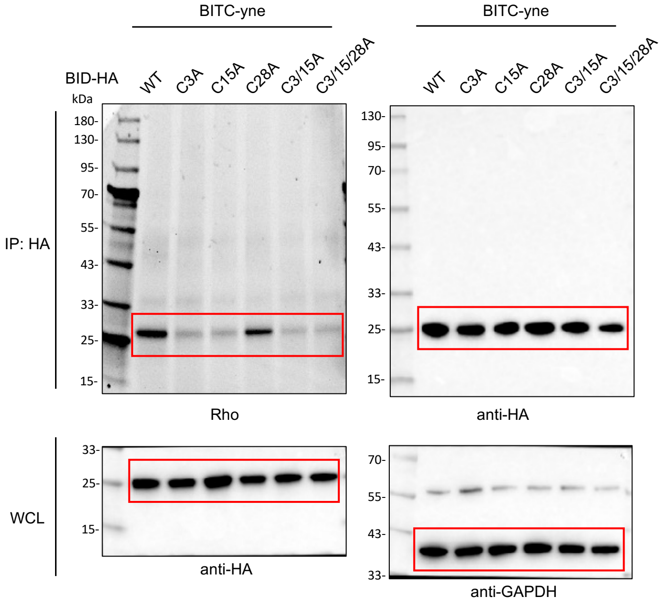

Figure 3d

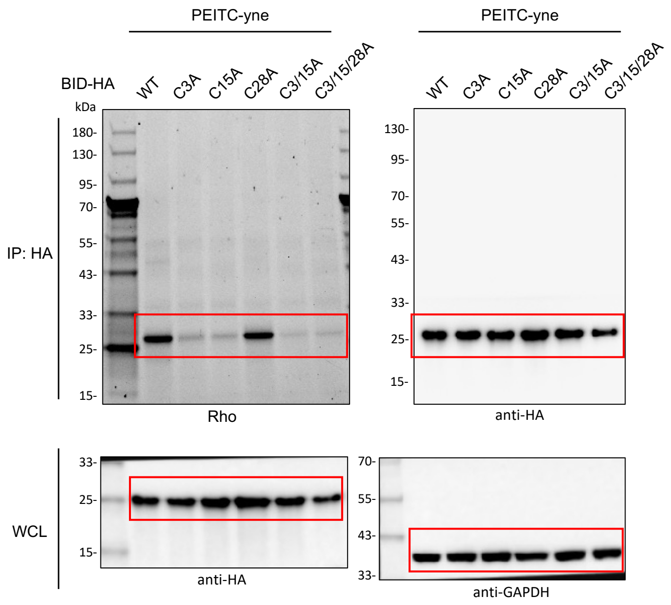

Figure 3f

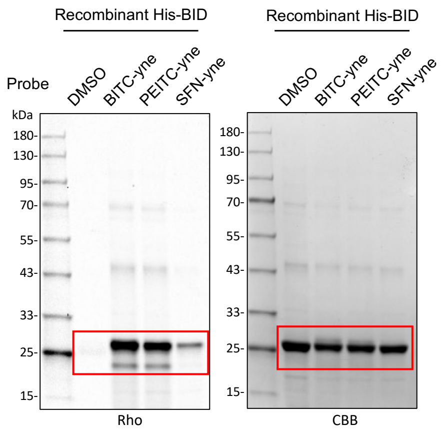

Figure 3e

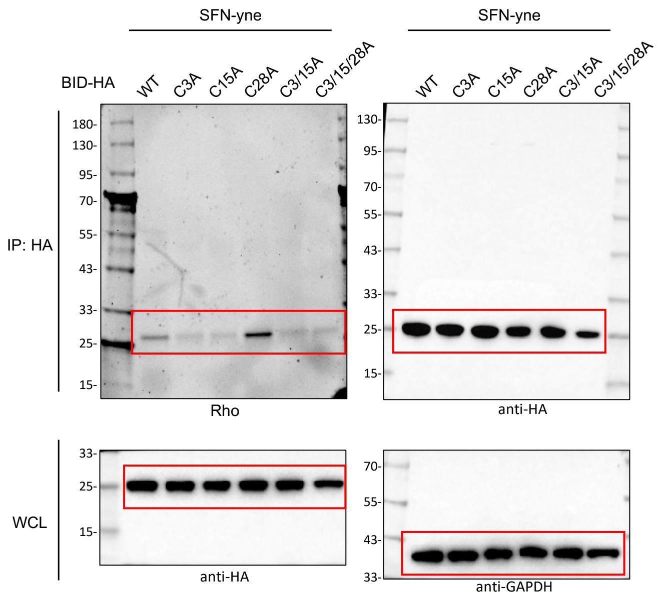

Figure 4a

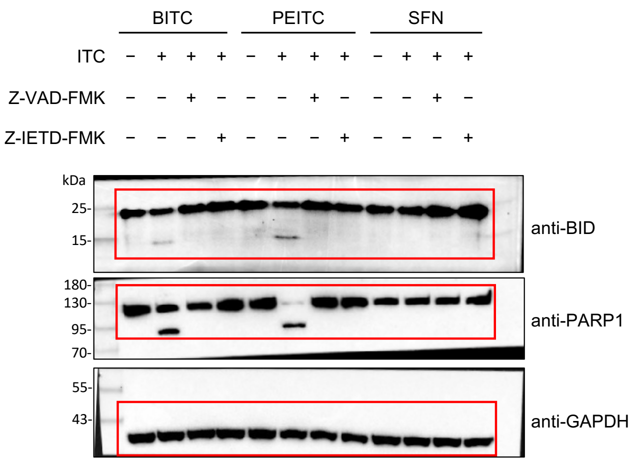

Figure 4d

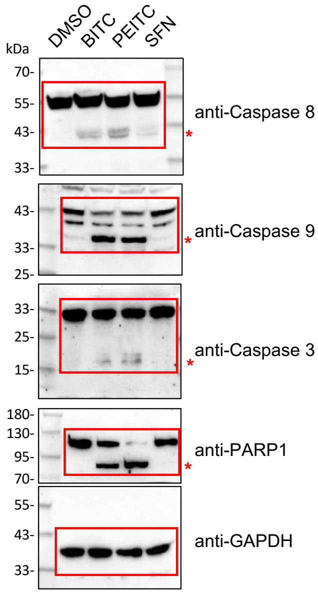

Figure 4b

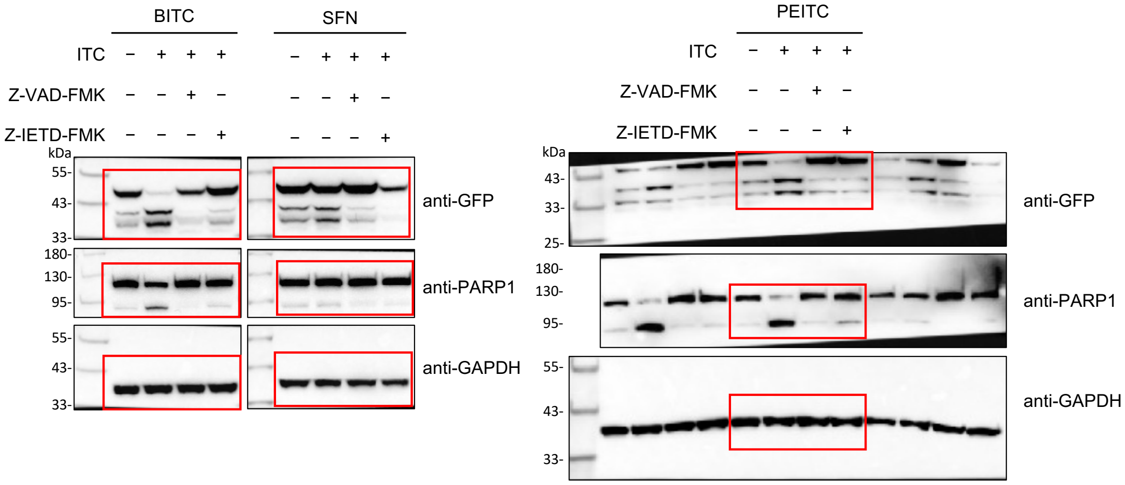

Figure 5b

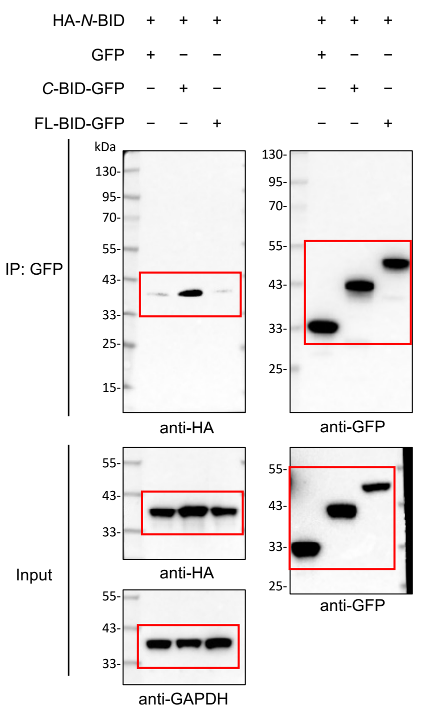

Figure 5e

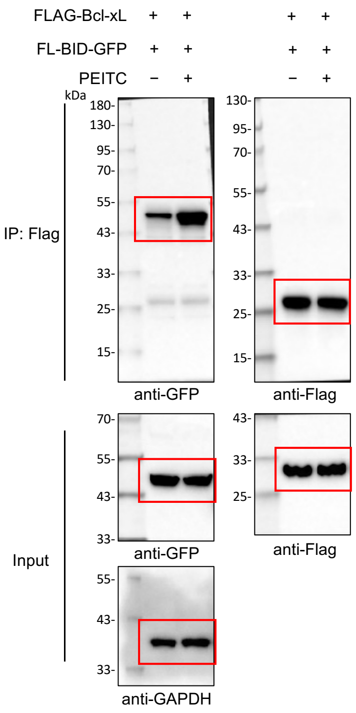

Figure 5f

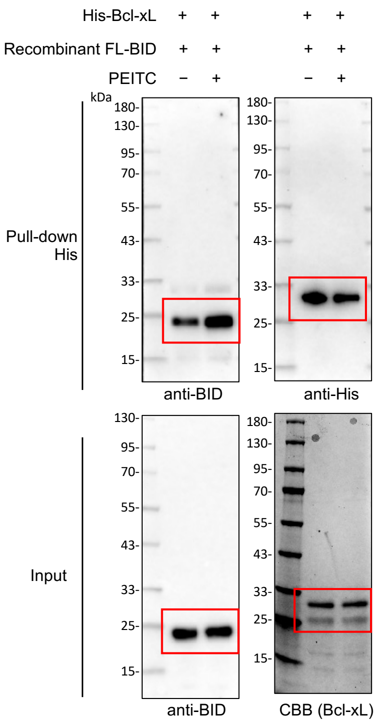

Figure 5c

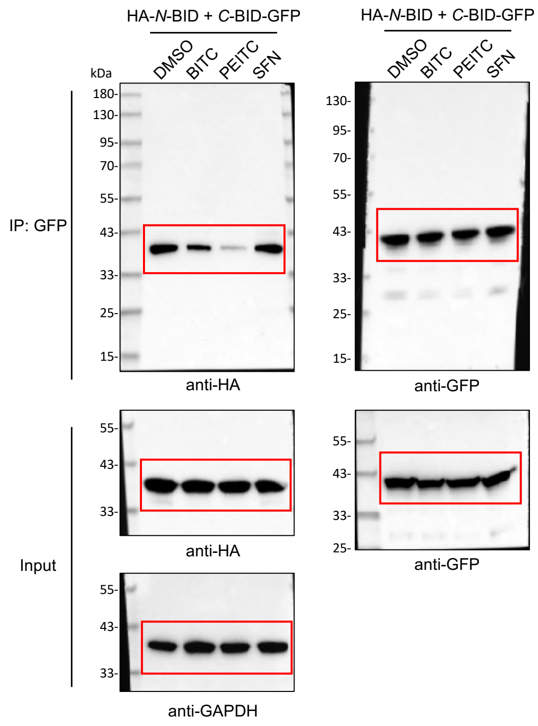

Figure 5d

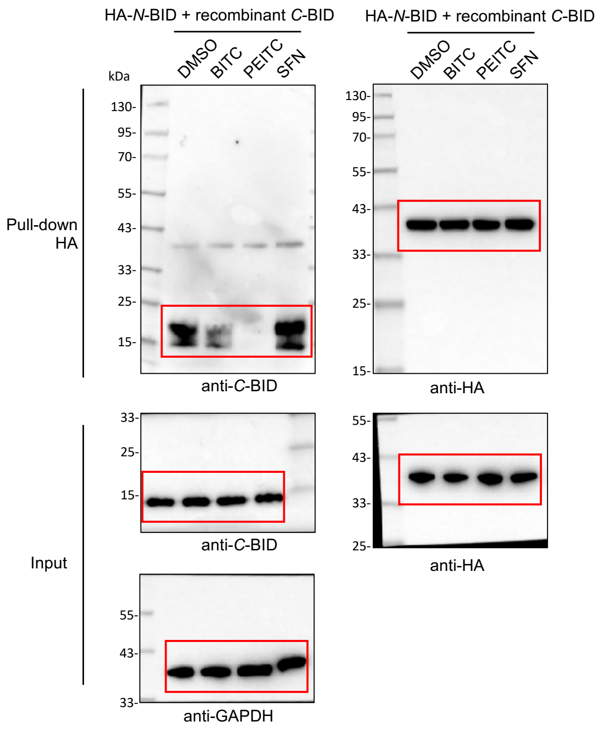

Figure 6a

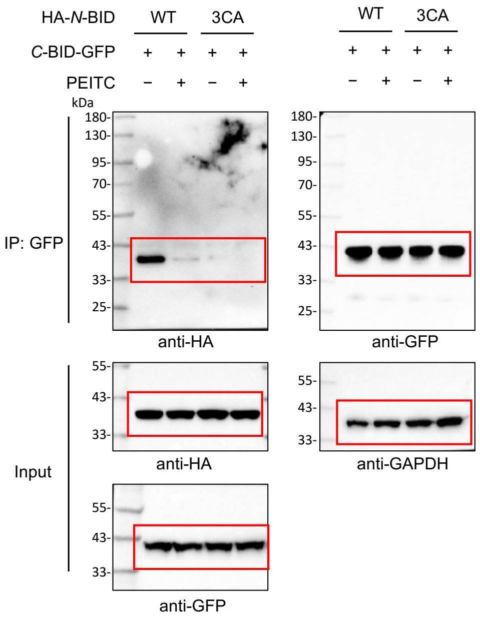

Figure 6b

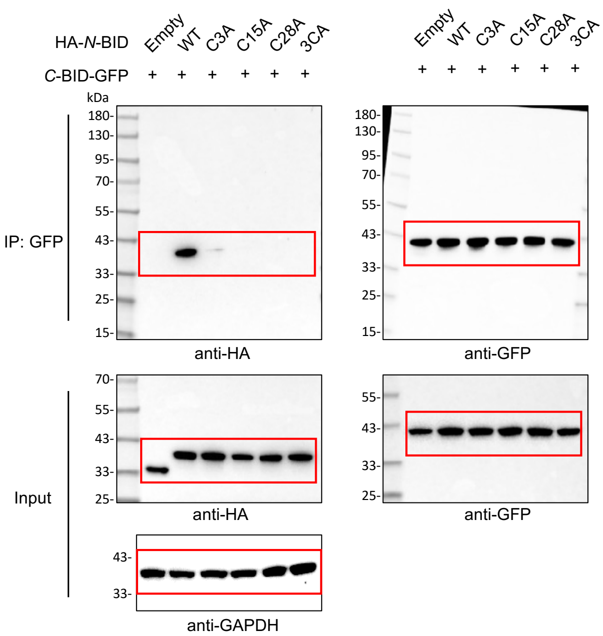

Figure 6c

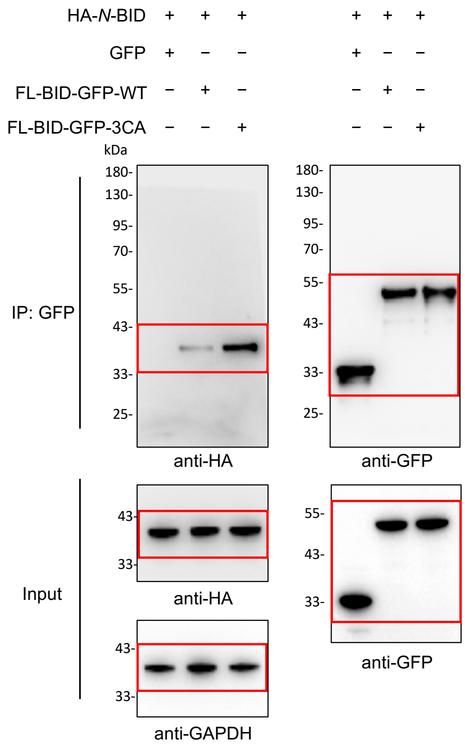

Figure 6d

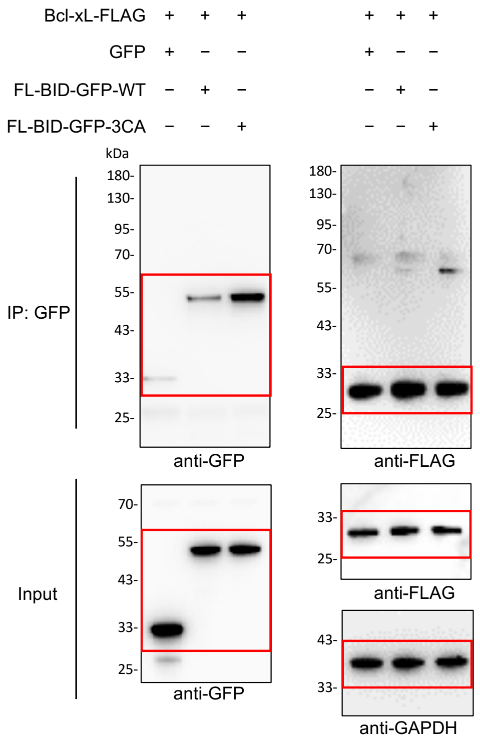

Figure 6e

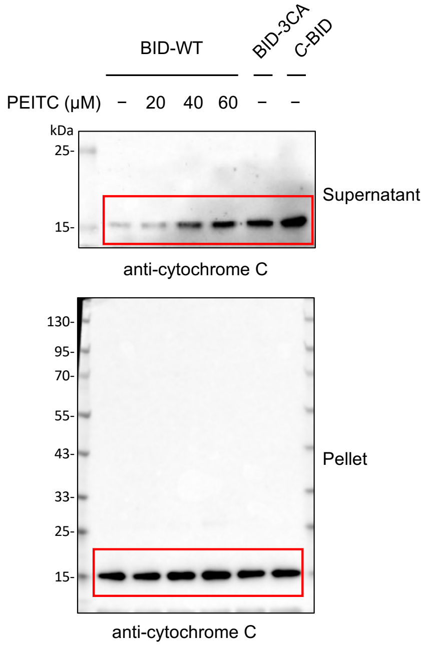

Figure S2a

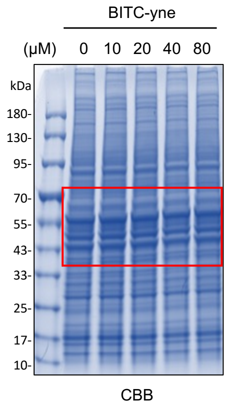

Figure S2b

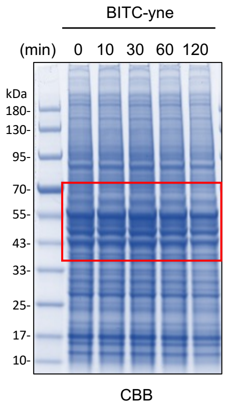

Figure S2c

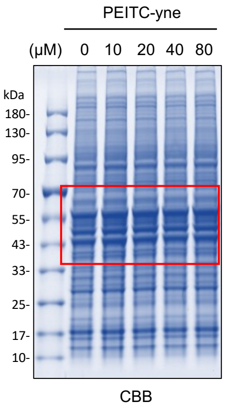

Figure S2d

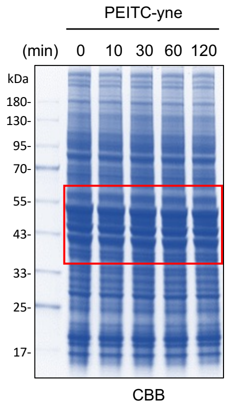

Figure S2e

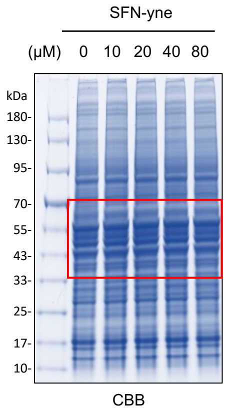

Figure S2f

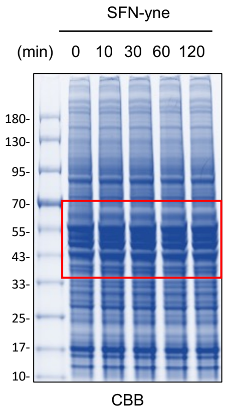

Figure S3

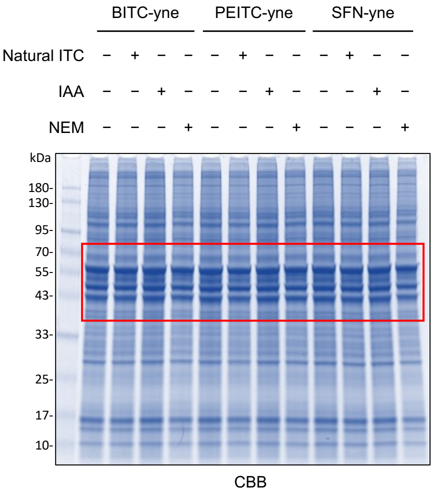

Figure S4b

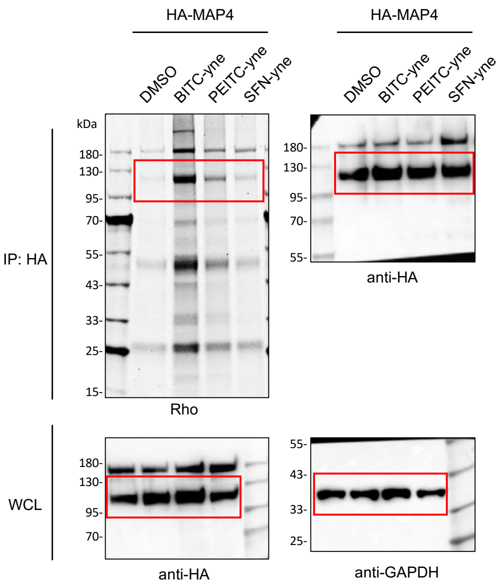

Figure S4c

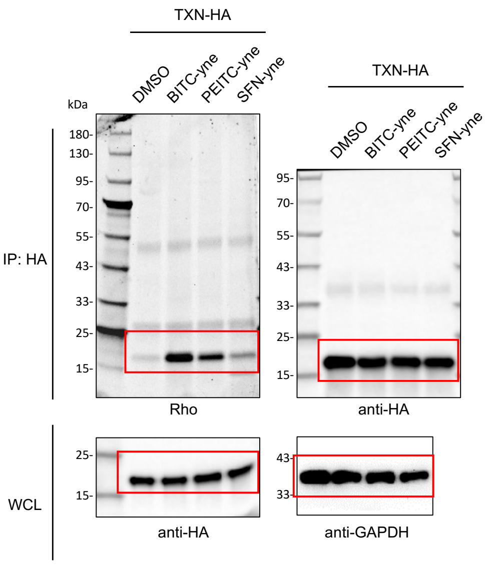

Figure S4d

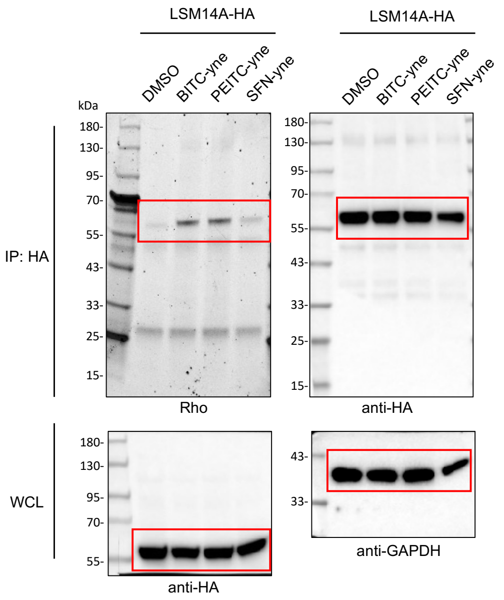

Figure S5

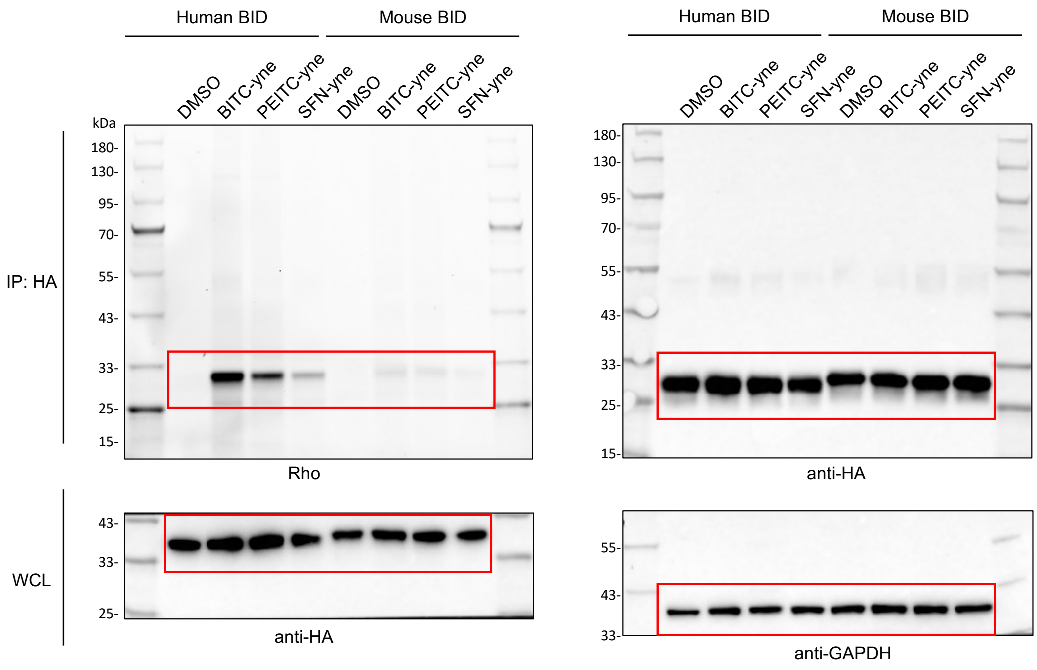

Figure S6a

Figure S6b

Figure S6c

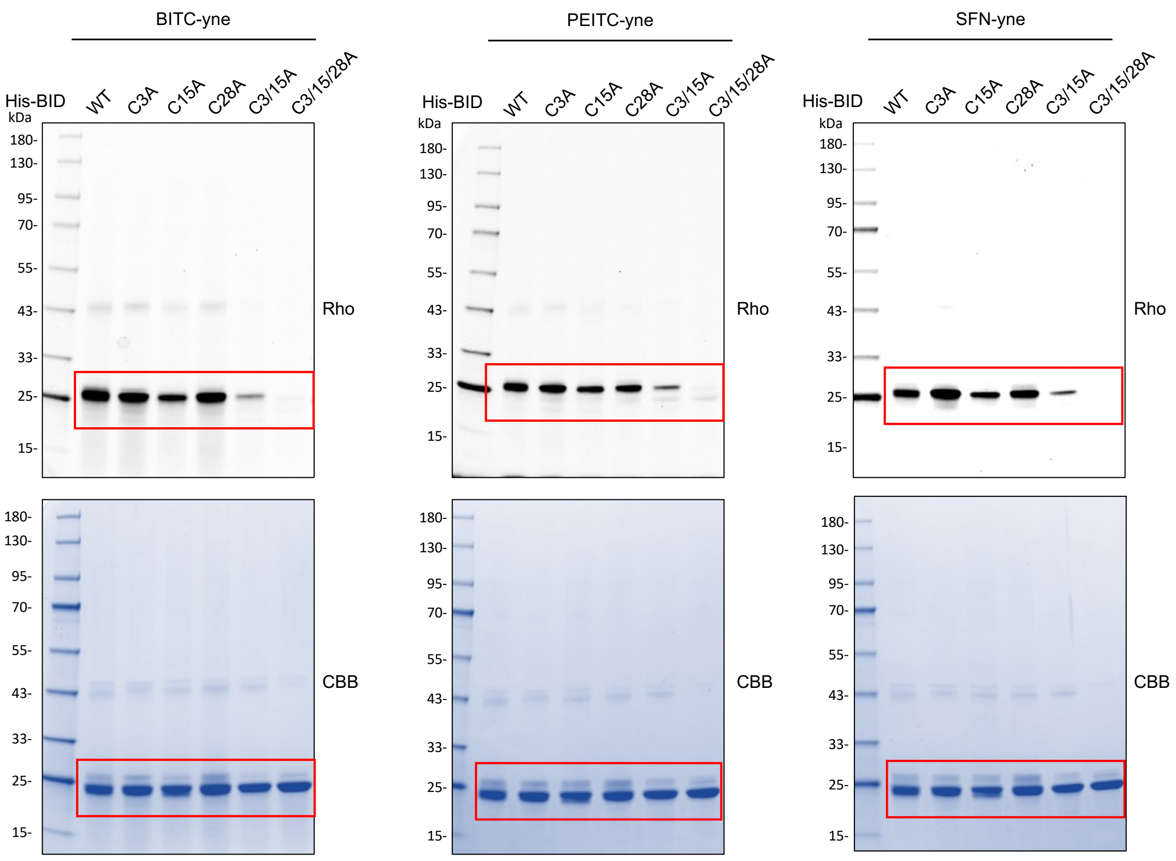

Figure S8a

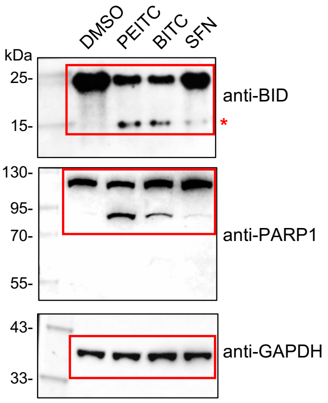

Figure S8b

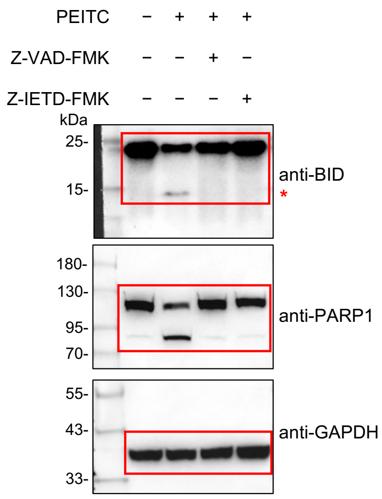

Figure S9

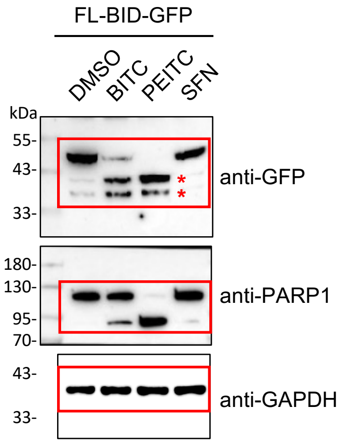

Figure S15a

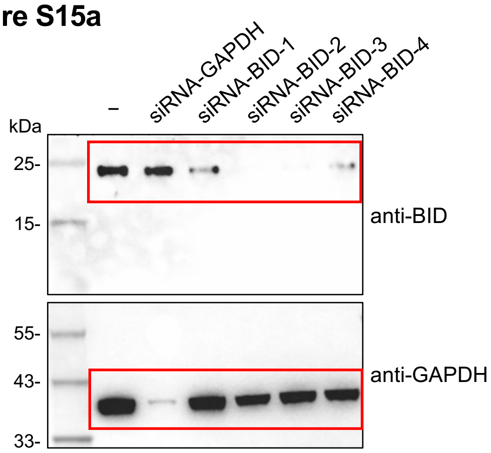

Figure S16

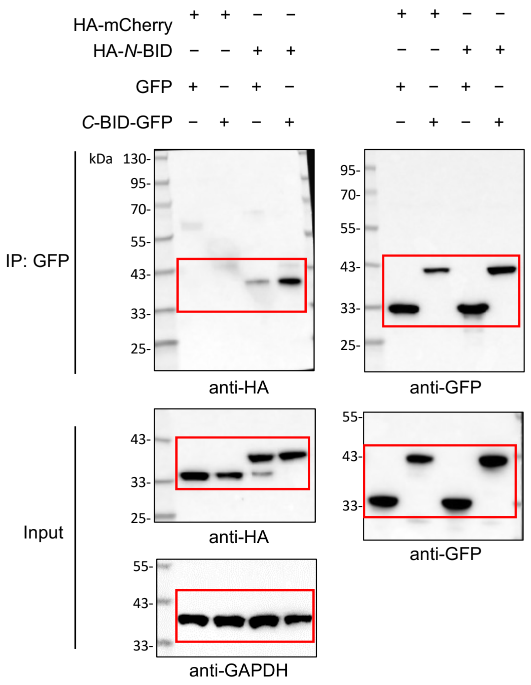

Figure S17

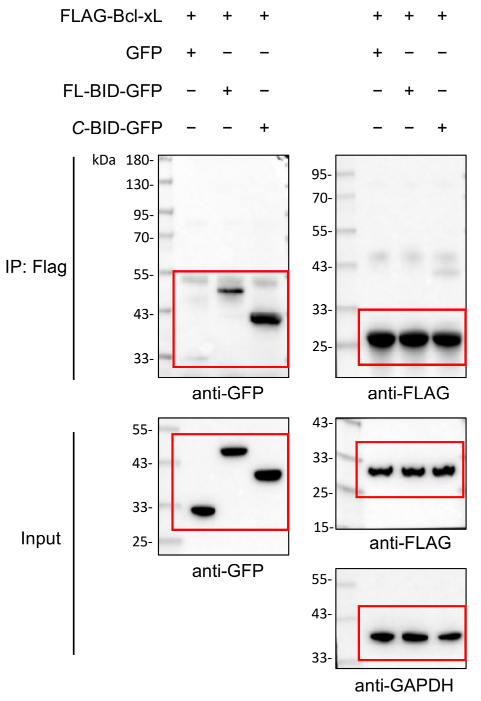

Figure S18a

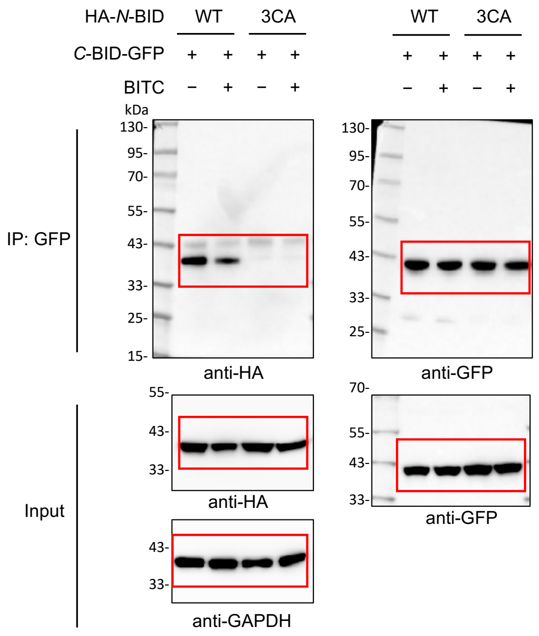

Figure S18b

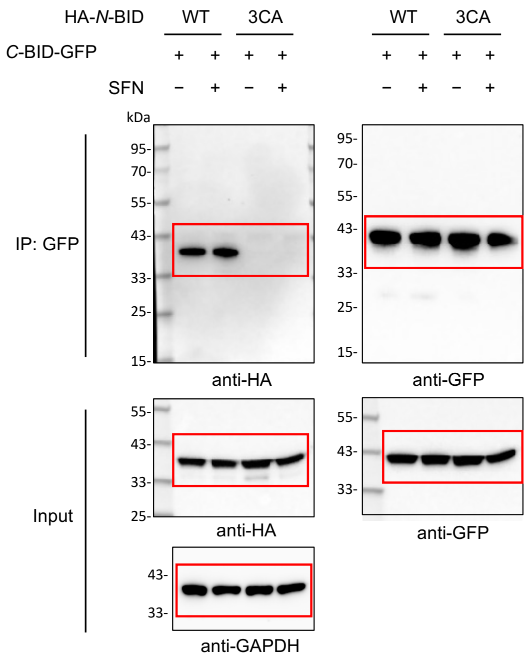

Figure S19a

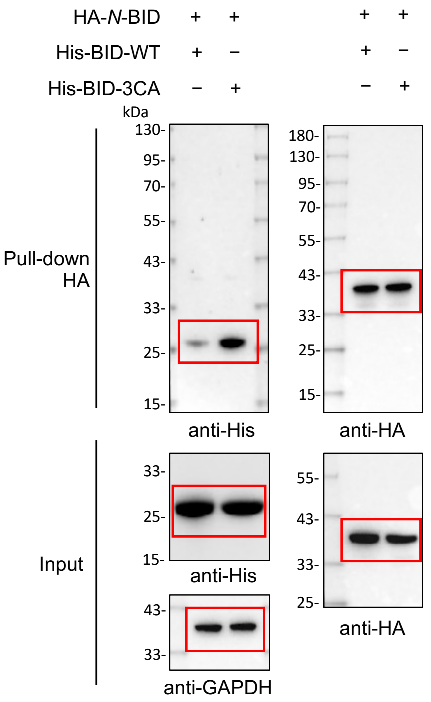

Figure S19b

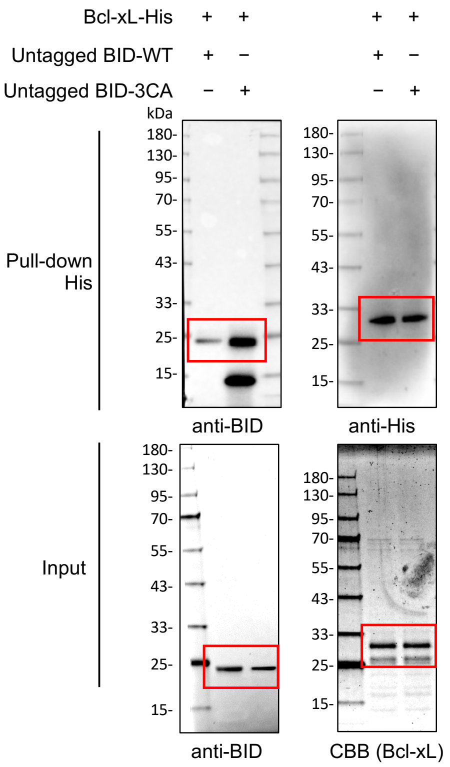

Supplement: Supplementary file 3 — Original Data [file 41420_2024_2225_MOESM3_ESM.pdf]
